# The epidemiology and transmissibility of Zika virus in Girardot and San Andres island, Colombia, September 2015 to January 2016

**DOI:** 10.2807/1560-7917.ES.2016.21.28.30283

**Published:** 2016-07-14

**Authors:** DP Rojas, NE Dean, Y Yang, E Kenah, J Quintero, S Tomasi, EL Ramirez, Y Kelly, C Castro, G Carrasquilla, ME Halloran, IM Longini

**Affiliations:** 1 Department of Epidemiology, University of Florida, Gainesville, FL, United States; 2 Department of Biostatistics, University of Florida, Gainesville, FL, United States; 4 Centro de Estudios e Investigacion en Salud, Fundacion Santa Fe de Bogota, Bogota, Colombia; 5 Secretaria Municipal de Salud, Girardot, Colombia; 6 IPS Universitaria, San Andres, Colombia; 7 Secretaria Departamental de Salud, San Andres, Colombia; 8 Department of Biostatistics, University of Washington, Seattle, WA, United States; 9 Vaccine and Infectious Disease Division, Fred Hutchinson Cancer Research Center, Seattle, WA, United States

## Abstract

Transmission of Zika virus (ZIKV) was first detected in Colombia in September 2015. As of April 2016, Colombia had reported over 65,000 cases of Zika virus disease (ZVD). We analysed daily surveillance data of ZVD cases reported to the health authorities of San Andres and Girardot, Colombia, between September 2015 and January 2016. ZVD was laboratory-confirmed by reverse transcription-polymerase chain reaction (RT-PCR) in the serum of acute cases within five days of symptom onset. We use daily incidence data to estimate the basic reproductive number (R_0_) in each population. We identified 928 and 1,936 reported ZVD cases from San Andres and Girardot, respectively. The overall attack rate for reported ZVD was 12.13 cases per 1,000 residents of San Andres and 18.43 cases per 1,000 residents of Girardot. Attack rates were significantly higher in females in both municipalities (p < 0.001). Cases occurred in all age groups with highest rates in 20 to 49 year-olds. The estimated R_0_ for the Zika outbreak was 1.41 (95% confidence interval (CI): 1.15–1.74) in San Andres and 4.61 (95% CI: 4.11–5.16) in Girardot. Transmission of ZIKV is ongoing in the Americas. The estimated R_0_ from Colombia supports the observed rapid spread.

## Introduction

First isolated in the Zika Forest of Uganda in 1947, Zika virus (ZIKV) is a flavivirus of the same genus as dengue virus and yellow fever virus. It is an arbovirus primarily transmitted by *Aedes aegypti* mosquitoes [1]. Although ZIKV has circulated in Africa and Asia since the 1950s, little is known about its transmission dynamics [2]. Recent outbreaks in Yap Island in Micronesia (2007), French Polynesia (2013), and other Pacific islands, including Cook Islands, Easter Island, and New Caledonia (2014), indicate that ZIKV has spread beyond its former geographical range [3–6]. In April 2015 ZIKV was isolated in the north-east of Brazil [7].

As of June 2016, around 500,000 Zika virus disease (ZVD) cases have been estimated in Brazil, and autochthonous circulation has been observed in 40 countries in the Americas. Further spread to countries within the geographical range of *Ae. aegypti* mosquitoes is considered likely [8].

Infection with ZIKV typically causes a self-limited dengue-like illness characterised by arthralgia, conjunctivitis, exanthema and low-grade fever [9]. While illness is believed to be mild or asymptomatic in ca 80% of the infections [10], an increase in rates of Guillain–Barre syndrome (GBS) has been observed during ZIKV outbreaks [8,11,12]. Furthermore, in October 2015, the Brazilian Ministry of Health reported a dramatic increase in cases of microcephaly in north-east Brazil where ZIKV had been circulating [13].

On the basis of the possible link between ZIKV, GBS and microcephaly, the World Health Organization (WHO) declared a public health emergency on 1 February 2016 [14,15].

**Figure 1:**
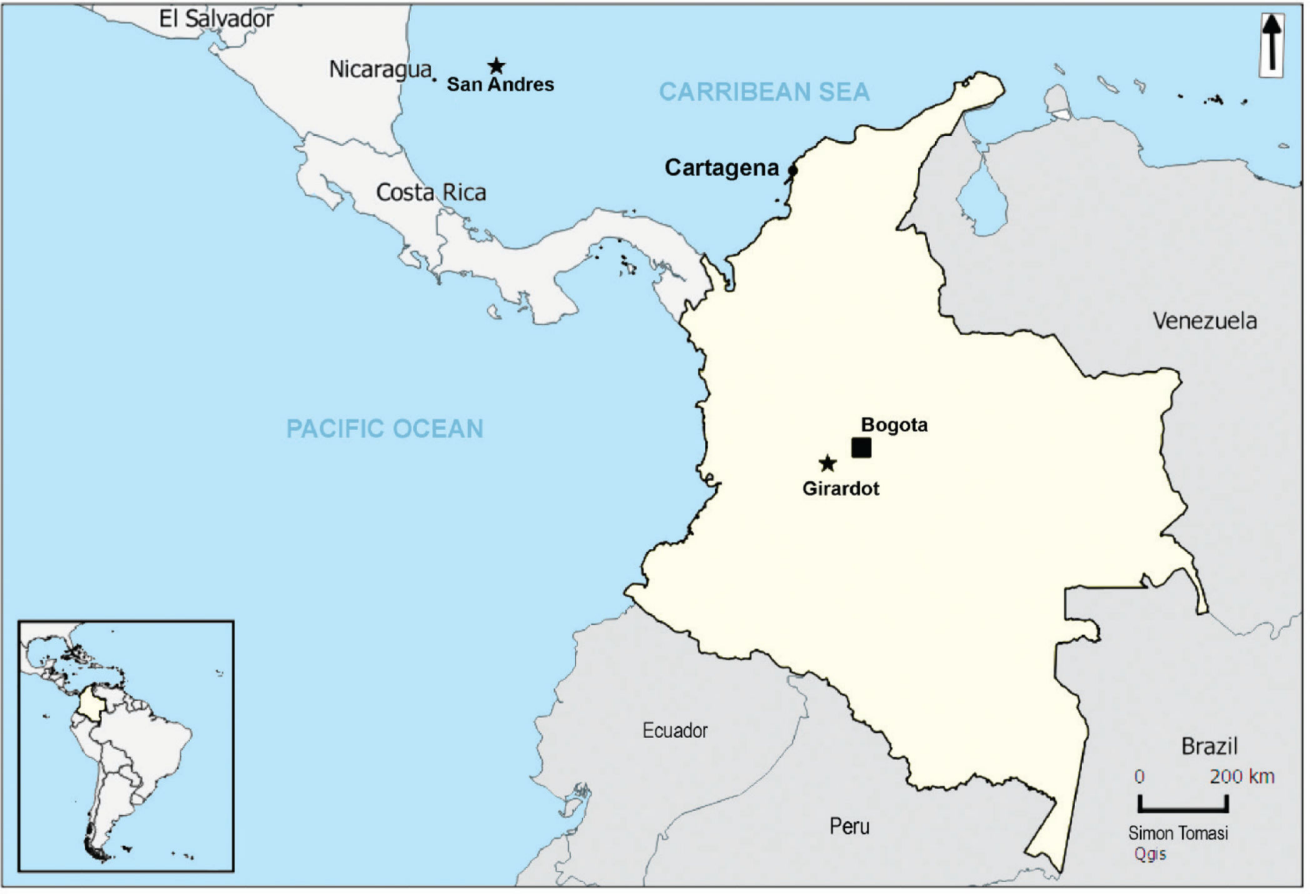
Location of the two Zika virus outbreak settings investigated, Colombia, September 2015–January 2016 Colombia figures in yellow on the map, with a dark square for the capital city Bogota. The two settings of Zika virus disease outbreaks investigated in this study are indicated by a star. On the map, the city of Cartagena is also shown, because in Colombia, Zika virus was first detected ca 10 km from this city, before spreading to other locations in the country.

In Colombia, the virus was first detected in mid-September 2015 in a municipality called Turbaco on the Caribbean coast. Turbaco is located 10.1 km from Cartagena (ca 20 min drive), a well-known commercial and tourism hub (Figure 1).

In October 2015, ZIKV spread through the central region of the country, appearing in areas infested with *Ae. aegypti* and with endemic dengue transmission and ongoing circulation of chikungunya virus (CHIKV) since 2014. By April 2016, Colombia had reported over 65,000 cases of ZVD, making it the second country most affected by ZIKV after Brazil [16,17]. Up to April 2016, 280 cases of neurological complications including GBS as well as seven deaths possibly associated with ZVD had been reported in Colombia [18]. As of April 2016, there have been four confirmed cases of ZIKV congenital syndrome in the country [17].

In this paper we describe local ZIKV outbreaks between September 2015 and January 2016 in Girardot and San Andres island, two different geographical areas in Colombia for which detailed epidemiological data are available. We conduct an investigation to define the epidemiological features of these outbreaks and to estimate the corresponding transmission parameters.

## Methods

### Settings

#### San Andres

San Andres is the largest island in a Colombian archipelago in the Caribbean Sea located ca 750 km north of mainland Colombia and 230 km east of Nicaragua (Figure 1). The island has an area of 27 km2, a population of 54,513 inhabitants across 13,652 households, and a population density of 2,932 habitants per km2 in 2010 [19,20]. Tourism is the most important economic activity in San Andres, with two high touristic seasons: June to July and December to January. The average temperature is 27.3 °C, and 80% of the total annual rainfall of 1,700 mm occurs during the heavy rainy season between October and December. The weather is humid subtropical with occasional hurricanes. The population in San Andres has two main ethnic groups: Afro-Colombians (17.5%) and Raizal (an ethnic group of mixed Afro-Caribbean and British descent) (39.2%) [20]. The most productive breeding sites of *Ae. aegypti* in San Andres are unprotected water containers located in the households. San Andres has experienced low dengue transmission (annual incidence rates <1%) since 1983. Since 1995, the frequency of dengue outbreaks increased every two to five years with a mean annual incidence of 0.43 cases per 1,000 inhabitants between 1999 and 2010 [19]. In 2014, CHIKV began circulating in San Andres, and that year it reached an annual incidence of 3.65 cases per 1,000 inhabitants [21].

#### Girardot

Girardot is a very central and well-connected municipality in continental Colombia. It is located 134 km (2 hours’ drive) from the capital city of Bogota, and it is a popular tourist destination for residents of Bogota (Figure 1). Girardot has 102,225 inhabitants across ca 23,000 households based on the most recent census from the National Statistics Department (NSD) [22], though the population can increase to 300,000 people during long weekends and high season holidays (June to July and December to January). Between 5 and 12 October 2015, a national beauty pageant in Girardot drew tourists from all regions in Colombia. Girardot is 289 m above sea level. The average temperature is 33.3 °C, and the relative humidity is 66%. The mean annual precipitation is 1,220 mm with a rainy season extending from May through October [23]. The most productive breeding sites of *Ae. aegypti* in Girardot are unprotected private water containers, such as water storage tanks used in the households during the dry and rainy seasons, while public spaces provide more breeding sites during the rainy season [24]. Girardot has experienced hyperendemic transmission of dengue since 1990 with simultaneous circulation of all four serotypes; the mean annual incidence was 5.72 per 1,000 inhabitants between 1999 and 2010 [19]. In late 2014, CHIKV started circulating in Girardot and that year it reached an annual incidence of 3.94 per 1,000 inhabitants, while in 2015 the annual incidence was 4.97 per 1,000 inhabitants [21,25].

### Case definition and laboratory analysis

We analysed surveillance data from nine local healthcare sites in San Andres and twenty-two local healthcare sites in Girardot, representing 100% of surveillance sites in both locations. Standardised case definitions used in both areas were defined by the Ministry of Health (MoH) and Colombian National Institute of Health (C-NIH) at the beginning of the ZIKV epidemic. According to these definitions, a suspected ZVD case is a person presenting with body temperature higher than 37.2 °C, maculopapular exanthema, and one or more of the following: arthralgia, headache, malaise, myalgia or non-purulent conjunctivitis and who lived or travelled to an area at risk for ZIKV transmission (usually below 2,000 m above sea level in Colombia) within 15 days of symptom onset. A laboratory-confirmed case is a suspected case with a ZIKV-positive reverse transcription-polymerase chain reaction (RT-PCR) result as determined by the C-NIH virology reference laboratory. ZIKV antibody testing was not done in Colombia due to high cross-reactivity with other endemic arboviruses. A clinically-confirmed case is defined by the Colombian authorities in the same way as a suspected case, except that the area of residence or travel within 15 days of symptom onset is an area with laboratory-confirmed ZIKV circulation [26].

Because the definition of a clinically-confirmed case in Colombia corresponded at the time of the study, to that of a probable case according to the WHO classification, we further refer to clinically-confirmed cases as probable cases in the context of this report [27].

At the start of the outbreaks in Girardot and San Andres, when local circulation of ZIKV had not yet been laboratory confirmed, only suspected cases were reported. Once the C-NIH confirmed the circulation of ZIKV in Girardot (on 27 January 2016, 3 months after the first local case report) and San Andres (on 22 October 2015, 45 days after the first local case report), the samples from suspected ZIKV cases were sent for laboratory confirmation if the respective cases fell into the risk groups defined by the C-NIH, including newborns and infants (age < 1 year), persons aged > 65 years, pregnant women, and individuals with comorbidities (e.g. diabetics, persons who were immunocompromised and/or with cardiovascular diseases) [23].

After ZIKV circulation was confirmed in the two areas, suspected cases whose acute samples tested positive were reclassified as laboratory-confirmed cases, while those with samples negative for ZIKV were reclassified as non-cases [26]. All reported suspected cases, who had not undergone laboratory testing were reclassified as probable cases [27].

### Data collection

The data in San Andres were collected initially using the C-NIH standard report form for dengue surveillance because from September up to October 2015 the outbreak in San Andres had an unknown aetiology. Once the C-NIH declared an alert on 14 October 2015 because ZIKV circulation had been observed in other areas of Colombia, reporting of ZVD became mandatory in the country, after which cases were reported by physicians at the healthcare sites using the standard report form for ZVD surveillance. The completeness of reporting is not known. We analysed a de-identified dataset based on place of residence with the following variables: age, sex, pregnancy status, date of symptom onset, date the case visited the healthcare facility, date the case was reported to the national surveillance system, and case type (suspected, laboratory confirmed, probable). Non-residents were excluded from the data [28].

### Statistical analysis

We calculated overall and age/sex-specific attack rates using population census data from NSD [22]. Surveillance data were analysed using R version 3.2.0 [29]. For descriptive results, categorical variables are presented as proportions and continuous variables by the median and interquartile range (IQR) or range. The relationship between attack rates and the variables age and sex was tested using log-linear models for case counts with age category (0–19 years-old, 20–49 years-old and >50 years-old), sex, and an interaction between age category and sex as independent variables, with population size as an offset.

To estimate the basic reproductive number R0 in each population, we used maximum likelihood methods to fit a chain-binomial model to daily incidence data [30]. The model assumes a mean serial interval of 22 days (time between successive cases in a chain of transmission); the serial interval takes into account the infectious period in humans, the extrinsic latent period in mosquitoes, the mean infectious period in the mosquito, and the mean incubation period in humans [4,9,31,32]. Underreporting is assumed to be high (only 10% of cases reported) at the start of the outbreak and full reporting is assumed to be achieved in four weeks after the outbreak begins to grow. With this assumption, we aimed to take into account the respective delays in the two sites, between the ZVD outbreak start and the confirmation by the C-NIH of circulation of ZIKV. R0 is the median effective reproductive number during the growth phase of the epidemic, after accounting for early underreporting (see supplementary materials online for additional details on the model: https://github.com/dprojas/Zika).

## Results

### San Andres

In San Andres, we identified 928 reported ZVD cases (Table 1). Of these cases, 52 (5.6%) were laboratory confirmed by RT-PCR on acute phase samples collected within five days of symptom onset, and 876 (94.4%) cases were probable.

**Figure 2:**
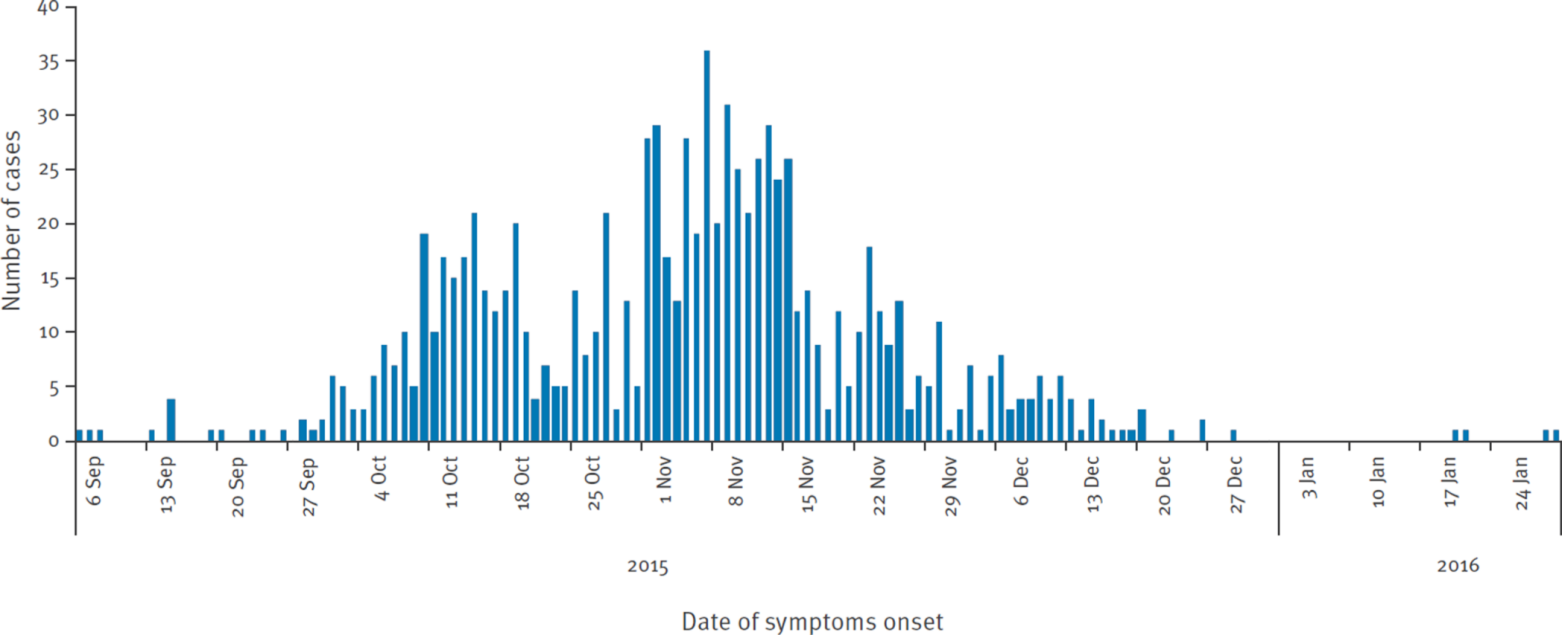
Daily Zika virus disease incidence in San Andres, Colombia, September 2015–January 2016 (n=928 cases) Cases include all reported cases, which were San Andres residents.

The dates of symptom onset among cases in San Andres ranged from 6 September 2015, to 30 January 2016 (Figure 2). Though the earliest case reported symptom onset on 6 September 2015, the local healthcare authorities did not receive laboratory confirmation of ZIKV until 22 October 2015. The distribution of this outbreak was bimodal. The first wave of the outbreak was before the C-NIH made an alert on 14 October 2015, about circulation of ZIKV in the country. The second wave started after the alert and the number of cases peaked in epidemiological week 45 (8 to 14 November), before the high tourist season started, and subsided in the last week of December. The second wave could be due to a reporting phenomenon.

The median time between symptom onset and visiting a healthcare facility was 4 days (IQR: 1–16).

Around 79% (733/928) of cases were reported to the national surveillance system on the same day that they visited the healthcare facility. The median age of reported ZVD cases in San Andres was 31 years-old (IQR: 15–47 years; range: 12 days–82 years). A total of 589 (63.5%) of the reported cases occurred in females. During the study period 238 dengue cases (incidence rate: 4.36 per 1,000 habitants) and 10 CHIKV cases (0.18 per 1,000 habitants) were reported in San Andres as expected in accordance with the trends and the historical data (data not shown).

**Figure 3:**
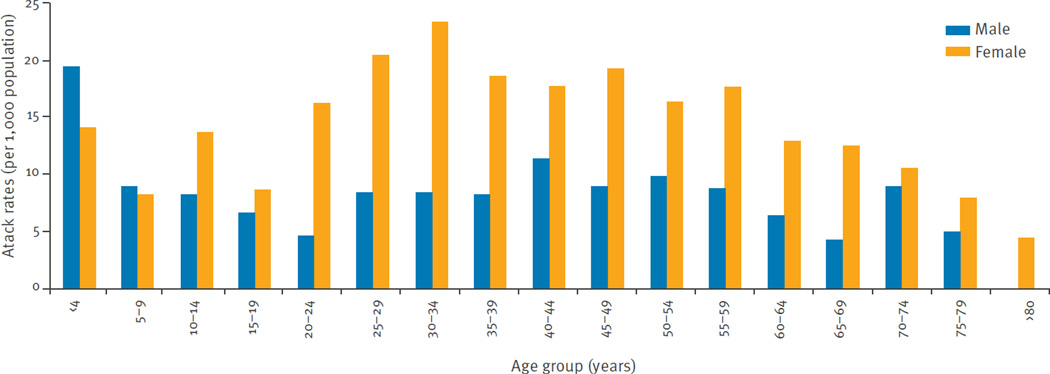
Age- and sex-specific Zika virus disease attack rates for San Andres, Colombia, September 2015–January 2016 (n=928 cases)

The overall attack rate for ZVD reported by local surveillance was 12.13 per 1,000 San Andres residents. The sex-specific attack rates were 15.34 per 1,000 females and 8.91 per 1,000 males; the difference was significant adjusting for age (p < 0.001). Cases occurred among all age groups, but the incidence of ZVD detected by local surveillance was highest among persons 20 to 49 years-old (Figure 3); there was significant heterogeneity across the age groups (p < 0.001). There was a significant interaction between age and sex (p < 0.001), consistent with the observation that attack rates were higher in females across all age groups 10 years-old and above, but lower for the younger age groups (Table 2).

Thirty-three pregnant women with ZVD were reported in San Andres and are being followed according to national guidelines [33,34]. By June 2016, twenty-eight of them had given birth with two probable cases of congenital ZIKV syndrome reported. There were eight neurological syndromes reported in San Andres, including GBS and meningoencephalitis attributed to ZIKV and among them one death was reported. The incidence rate of neurological syndromes among ZVD cases in San Andres is 8.6 per 1,000 cases.

### Girardot

In Girardot, we identified 1,936 reported ZVD cases (Table 1). Of these cases, 32 (1.7%) were laboratory confirmed by RT-PCR on acute phase samples collected within five days of symptom onset and 1,904 (98.3%) were probable.

**Figure 4:**
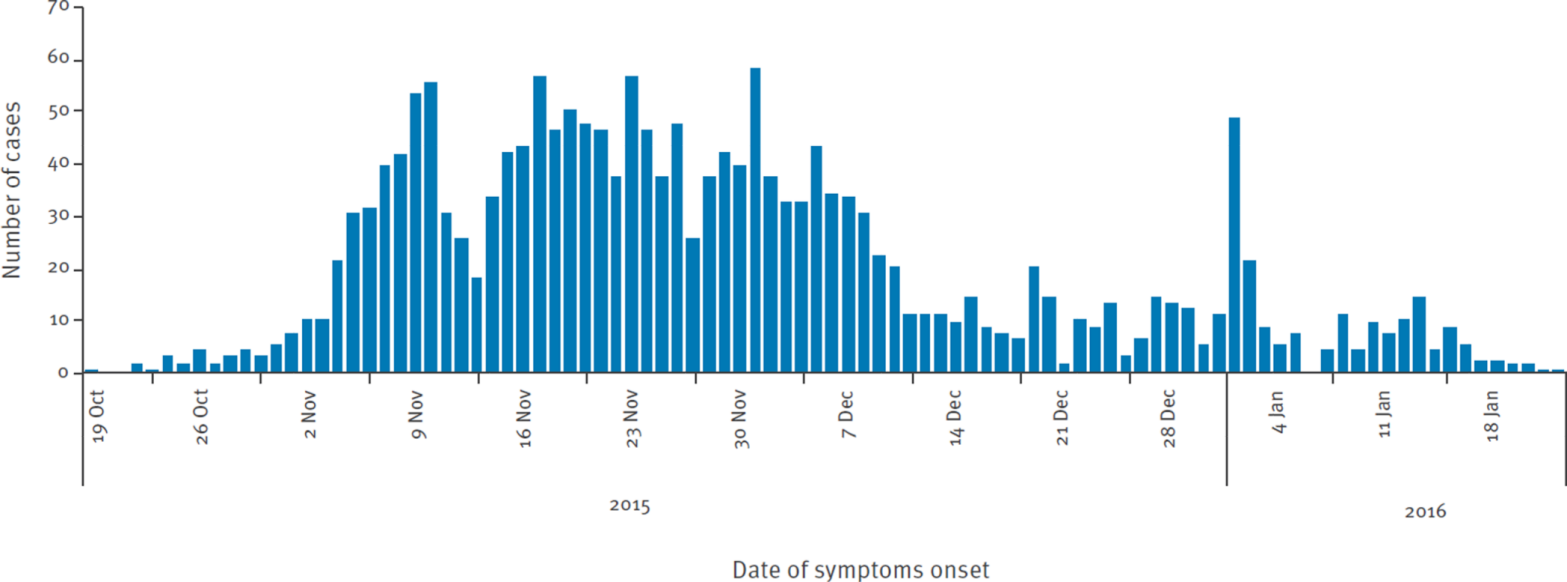
Daily ZVD incidence for Girardot, Colombia, October 2015–January 2016 (n=1,936 cases)

The date of symptom onset among cases in Girardot ranged from 19 October 2015 to 22 January 2016 (Figure 4). The first suspected case was reported on 23 October 2015, 19 days after the beauty pageant event started, with laboratory confirmation obtained on 27 January 2016. The number of cases peaked in epidemiological week 48 (29 November to 5 December) before the end-of-the-year tourist season, and subsided in early January.

The median time between symptom onset and visiting a healthcare facility was 1 day (IQR: 1–2 days). Around 89% (755/1,936) of cases were reported to the national surveillance system on the same day they visited the healthcare facility. The median age of confirmed ZVD cases was 34 years-old (IQR: 24–46 years; range: 15 days–92 years). A total of 1,138 (58.8%) cases were female. During the study period 75 dengue cases (incidence rate: 0.73 per 1,000 habitants) and 200 CHIKV cases (1.95 per 1,000 habitants) were reported in Girardot as expected in accordance with the trends and the historical data (data not shown).

**Figure 5:**
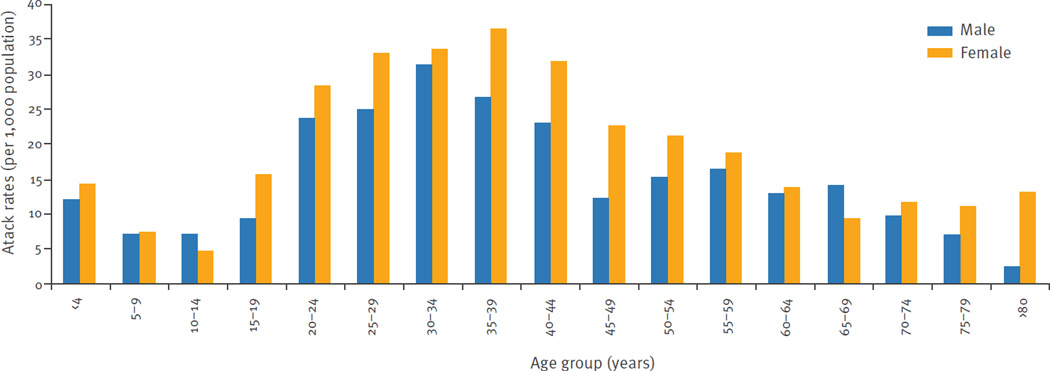
Age- and sex-specific Zika virus disease attack rates for Girardot, Colombia October 2015–January 2016 (n=1,936 cases)

The overall attack rate for confirmed ZVD detected by local surveillance was 18.43 per 1,000 Girardot residents. The sex-specific attack rates were 20.53 per 1,000 females and 16.07 per 1,000 males; the difference was significant adjusting for age (p < 0.001). Cases occurred among all age groups, but the incidence of ZVD detected by local surveillance was highest among persons 20 to 49 years-old (Figure 5); there was significant heterogeneity across the age groups (p < 0.001). Attack rates were higher in females in all age groups except in those 10 to 14 and 65 to 69 years-old; there was no significant interaction between age and sex (p = 0.20) (Table 2).

Sixteen pregnant women with ZVD were reported in Girardot and are being followed according to national guidelines [33,34]. By June 2016, twelve of them had given birth with no complications or microcephaly reported. Nine cases with GBS have been reported after an initial suspected ZIKV infection; laboratory-confirmation of ZIKV is pending. There were no deaths attributed to ZIKV. The incidence rate of neurological syndromes among ZVD cases in Girardot is 4.6 per 1,000 cases.

### Basic reproductive number calculations

**Figure 6:**
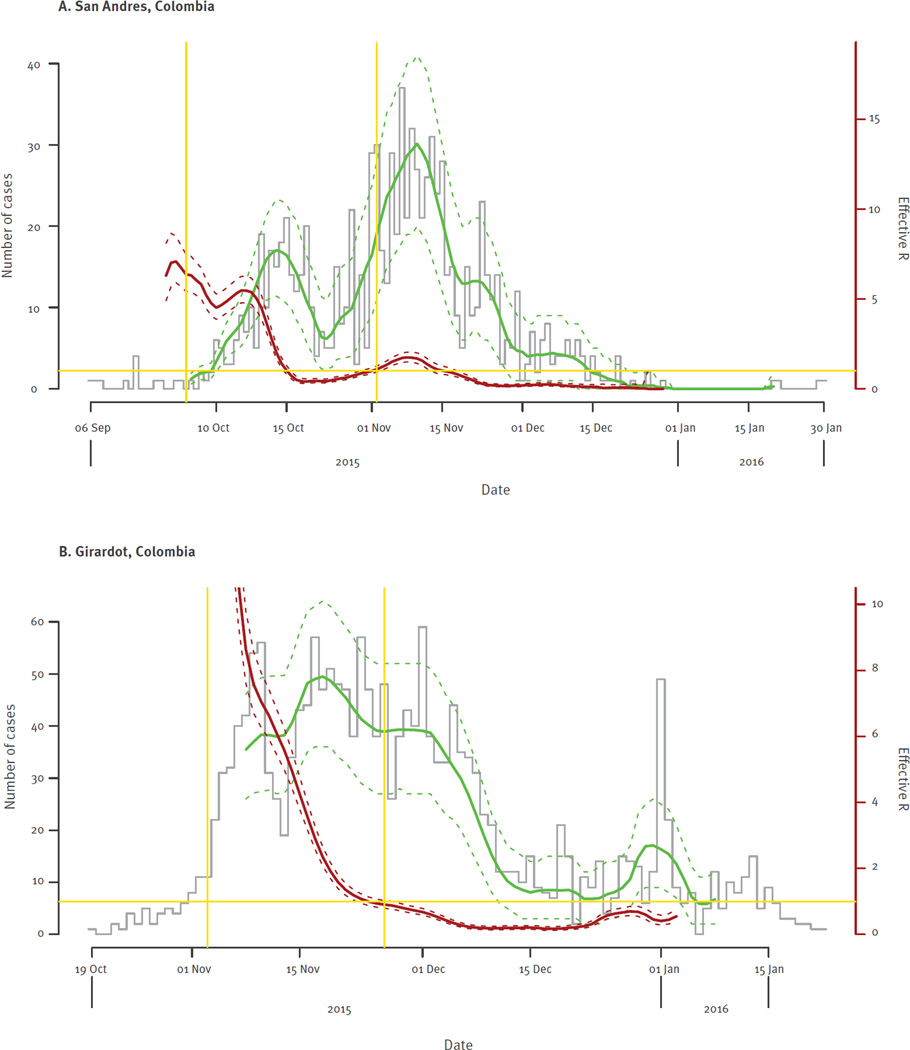
Estimates of effective R (red) and model-fitted daily case numbers (green) for outbreaks of Zika virus disease in Colombia, September 2015–January 2016 (n=2,864 cases CI: confidence interval; R0: basic reproductive number. (A) Estimates of effective R (red) and model-fitted daily case numbers (green) for the outbreak of ZVD in San Andres, Colombia. The proportion of cases reported is assumed to increase linearly from 10% on and before 30 September 2015, to 100% in 4 weeks. Dashed curves (both red and green) are conservative 95% CIs. Histogram in grey shows the epidemic curve. The horizontal yellow line indicates the reference value of 1. The two vertical yellow lines indicate the time interval used for the estimation of R0. (B) As (A) for Girardot, Colombia. The proportion of cases reported increases on 19 October 2015.

Daily incidence data were used to estimate R0. The estimated R0 for the Zika outbreak in San Andres was 1.41 (95% confidence interval (CI): 1.15–1.74), and the R0 in Girardot was 4.61 (95% CI: 4.11–5.16) (Table 2 and Figure 6). Odds ratios for sex and age effects were obtained from the likelihood model, indicating increased odds of transmission among females and adults aged 20 to 49 years-old in both San Andres and Girardot (Table 2).

The estimation procedure was also applied to daily incidence data from a published outbreak in Salvador, Brazil, that occurred between 15 February 2015, and 25 June 2015; 14,835 cases were reported with an overall attack rate of 5.5 cases per 1,000 Salvador residents [7]. The estimated R0 of the Zika outbreak in Salvador, Brazil was 1.42 (95% CI: 1.35–1.49).

Sensitivity analyses are reported in the supplementary online materials (https://github.com/dprojas/Zika), including varying the incubation period in humans, the infectious period in humans, the infectious period in mosquitoes, the duration of underreporting, and the level of underreporting at the start of the outbreak.

## Discussion

We report surveillance data on ZIKV outbreaks in two areas in Colombia between September 2015 and January 2016. The first area, San Andres, is a small, densely populated island that is relatively isolated from continental Colombia. The second area, Girardot, is a typical moderately sized Colombian municipality. Both regions have endemic transmission of dengue and experienced recent outbreaks of CHIKV. We describe key epidemiological features of the ZVD outbreaks and estimate R0 from daily incidence data.

The overall attack rates for ZVD as detected by local surveillance were 12.13 cases per 1,000 residents of San Andres and 18.43 cases per 1,000 residents of Girardot. These attack rates are similar to those reported from Yap Island (14.3 per 1,000) [3] but higher than those reported in Salvador, Brazil (5.5 per 1,000) [7]. In both areas, significantly higher attack rates are observed among women, especially those of child-bearing age. The Colombian government issued an epidemiological alert in December 2015 to actively search for pregnant women with ZVD-like symptoms in areas with active transmission [33,34]. This effort may partially explain the findings, though differences in sex-specific attack rates persist when only cases occurring before December are considered. These results could be explained by male-to-female sexual transmission of ZVD, which is consistent with higher attack rates in females beyond child-bearing age. Given recent evidence from Brazil, in areas with ZIKV transmission, interventions aimed at preventing sexual-transmission of ZIKV to women are necessary because this mode of transmission could have a substantial influence on the overall dynamics of ZIKV epidemics [35–37]. Cases occurred in all age groups, but the most affected age group was 20 to 49 year of age, similar to previously published outbreaks in Yap Island, Micronesia, and in Salvador, Brazil [3,7]. As the population was fully susceptible to ZIKV transmission before the outbreaks, it is expected that all age groups would be affected.

Forty-nine pregnant women with ZVD were reported from San Andres and Girardot. These women are being followed according to national guidelines [33,34] with two probable cases of congenital ZIKV syndrome reported from San Andres to the national authorities for analysis. Seventeen cases of neurological syndrome, including GBS and ZIKV-associated meningoencephalitis, were identified, similar to reports from French Polynesia and Brazil [12,38]. Laboratory-confirmation of these cases is challenging because neurological symptoms generally appear two weeks after acute symptoms [39] at which time ZIKV diagnosis by RT-PCR is not possible and serological tests are unreliable because of cross-reactivity with dengue [40,41]. As ZIKV can be detected in urine longer than in serum [42], using urine samples to confirm ZIKV in GBS cases may be an alternative [43]. These challenges underscore the need for reliable diagnostic tests that can detect ZIKV after the viraemic period.

In each area of this study, daily incidence data were used to estimate R0. Our estimated R0 for the ZVD outbreak in San Andres was 1.41 (95% CI: 1.15–1.74), and the R0 for Girardot was 4.61 (95% CI: 4.11–5.16). Applying the same methods with previously published data, we estimated that the R0 for ZIKV in Salvador, Brazil, was 1.42 (95% CI: 1.35–1.49) [7]. We consider the estimate from San Andres to be the most reliable because it is a small, densely populated island and the outbreak occurred before the national epidemiological alert, while Girardot has a higher risk of importation because the population fluctuates during weekends and holidays. The relative magnitudes of R0 are consistent with the higher dengue transmission historically observed in Girardot vs San Andres [19].

Estimates of R0 in ZIKV are not widely available, though reports suggest an R0 of 4.3 to 5.8 in Yap Island and R0 of 1.8 to 2.0 in French Polynesia [44]. A recent manuscript considering the French Polynesian outbreak reported a range from 1.9 to 3.1 [45].

Relatively few cases were laboratory confirmed. One limitation of this study is that the majority of cases were probable, and the symptoms could be caused by other aetiologies such as dengue or CHIKV. Nonetheless, in the field we have observed that the diseases have different clinical manifestations. Dengue appears to coincide with high fever (> 38.5 °C), headaches, myalgia, and generalised pain. CHIKV is associated with joint pain and arthritis, and ZVD is associated with a very mild, low-grade fever (38 °C) or no fever, rash, and no generalised pain. This report only includes symptomatic cases who attended a healthcare facility and were captured by the surveillance systems. ZIKV usually causes a relatively mild illness lasting several days, and around 80% of infections are currently believed to be asymptomatic, so we are likely missing many mild or asymptomatic cases [10]. We also do not have a reliable estimate of underreporting at the study sites. Early underreporting seemed to be especially apparent in the Girardot outbreak compared with San Andres given that the circulation of ZIKV was not confirmed until January, 2016, and the sharp increase in cases in Girardot observed may be due to increased public awareness of the disease. This phenomenon can result in an overestimate of R0.

Well-designed studies can provide valuable insight. Phylogenetic analyses of circulating ZIKV strains will be critical for understanding whether mutations in the viral genome are associated with an increased severity of disease, as manifested by microcephaly and GBS in this outbreak. Household studies can allow for more accurate estimation of transmission dynamics and enhance understanding of asymptomatic infection. Studies are required to understand the interactions between ZIKV, dengue, CHIKV, and other co-circulating arboviruses and their impact on disease. It is also necessary to increase surveillance of neurological syndromes associated with ZVD, such as GBS and encephalitis.

The evidence for a causal relationship between ZIKV and microcephaly is strengthening [46–48]. Recent evidence from the French Polynesia outbreak suggests an estimated number of microcephaly cases possibly associated with ZIKV infection is around one per 100 women infected in the first trimester [49]. Currently the Colombian Government is following a cohort of pregnant women that reported ZVD-like symptoms anytime during their pregnancy. Those who are detected during the acute phase are being diagnosed with ZIKV RT-PCR. All women will be followed until the end of pregnancy, and the fetus will be evaluated during pregnancy, with a subsequent post-natal follow-up of twelve months [17]. The prospective collection of data through this and other similar national cohorts will be essential for assessing causality, determining risk factors, and estimating rates of birth defects.

The results of this and other reports conclude that transmission of ZIKV may be widespread. Vector control has had limited success in controlling other arboviruses, such as dengue. A safe and efficacious vaccine, especially for women of child-bearing age, may be needed to reduce the disease burden.

## Supplementary Material

Supplemental Materials

## Figures and Tables

**Table 1:** Characteristics of reported cases of Zika virus disease in two areas of Colombia, September 2015–January 2016

Areas	San Andres	Girardot
Total number of cases	928	1,936
Laboratory confirmed cases n (%)	52 (5.6%)	32 (1.7%)
Probable cases n (%)	876 (94.4%)	1,904 (98.3%)
Female n (%)	589 (63.5%)	1,138 (58.8%)
Median age in years (IQR)	31 (15–47)	34 (24–46)
Median time in days to visit healthcare facility from symptom onset (IQR)	4 (1–16)	1 (1–2)

IQR: interquartile range.

**Table 2:** Estimates of basic reproductive number (R_0_), sex-specific odds ratios (OR) and age-specific OR for transmission of Zika virus disease in San Andres and Girardot, Colombia, September 2015–January 2016

Parameter Estimate (95%CI)		San Andres	Girardot
		Estimate (95%CI)	
R_0_		1.41 (1.15–1.74)	4.61 (4.11–5.16)
OR sex	Male	Reference	Reference
	Female	1.71 (1.50–1.95)	1.28 (1.17–1.40)
OR age in years	20–49	Reference	Reference
	0–19	0.86 (0.74–0.99)	0.37 (0.33–0.42)
	> 50	0.74 (0.63–0.88)	0.46 (0.41–0.52)

CI: confidence interval.
