# Supplementary material for: The epidemiology and transmissibility of Zika virus in Girardot and San Andres island, Colombia, September 2015 to January 2016
Source: Euro Surveill. Author manuscript; Available in PMC 2016 Nov 26. (PMC5124348; doi:10.2807/1560-7917.ES.2016.21.28.30283)
Supplement: Supplemental Materials [file NIHMS831071-supplement-Supplemental_Materials.pdf]

# The Epidemiology and Transmissibility of Zika Virus in Girardot and San Andres, Colombia

Supporting Online Material

April 22, 2016

# Contents

**1 Natural history of Zika in Human and Mosquito** **3**

**2 Estimation of effective reproductive numbers($R_t$) and basic reproductive number ($R_0$)** **5**

2.1 $R_0$ results . . . . . . . . . . . . . . . . . . . . . . . . . . . . . . . . . . . . 10

2.2 Sensitivity analyses for under-reporting . . . . . . . . . . . . . . . . . . . . . . . 22

# 1 Natural history of Zika in Human and Mosquito

Little is known about the natural history of Zika virus disease (ZVD). Below we define the incubation period within humans, the infectious period within humans, the extrinsic latent period in mosquitoes, and the infectious period in mosquitoes. The convolution of these determines a potential range for the serial interval of Zika, where the serial interval is defined as the mean time between successive cases of ZVD.

The incubation period in a Zika virus (ZIKV) infected human case, which is the time from infection to the onset of illness, is assumed to follow a discrete distribution $\Pr(\delta = l) = \alpha_l$, subject to $\sum_{l=\delta_{min}}^{\delta_{max}} \alpha_l = 1$, where $\delta$ represents the duration of the incubation period in days, and $\delta_{min}$ and $\delta_{max}$ are the lower and upper bounds of the incubation period, respectively. Table S1 describes three sensitivity settings for the incubation period in humans, all of which have range 3 to 7 days: (1) the short setting has mean 4.3 days, (2) the medium setting has mean 5 days, and (3) the long setting has mean 5.7 days.

An infected human $i$ remains infectious for $D$ days from the date of illness onset (including the date of illness onset). The infectiousness level is constant over the infectious period. Let $D_{min}$ and $D_{max}$ be the lower and upper bounds of $D$, respectively. $D$ is uniformly distributed between $D_{min}$ and $D_{max}$. The probabilities of this person being infectious on day $j$ since infectiousness onset is denoted by $\gamma_j = P(D \geq j), j = 1, \ldots, D_{max}$. As symptoms caused by ZIKV usually resolve in about a week [3], we choose 4 to 8 days as the range of the mean infectious period. Table S1 describes three sensitivity settings for the infectious period in humans: (1) the short setting has a range of 2 to 6 days with mean 4 days, (2) the medium setting has a range of 4 to 8 days with mean 6 days, and (3) the long setting has a range of 6 to 10 days with mean 8 days.

Given that a mosquito bites an infectious case, we assume that biting occurs equally likely

over the human's infectious period, after which a fixed extrinsic latent period of $\delta^\star = 10$ days follows [4].

The natural time span of an adult mosquito is around 30 days [2, 5]. It is likely that, once infected and the extrinsic latent period has passed, the virus reproduces in a mosquito's salivary gland throughout its life. Similar to the infectious period of humans, we assume the infectious period of an infected mosquito, denoted by $D^\star$ days, is also uniformly distributed within the given bounds, $D^\star_{min}$ and $D^\star_{max}$. The corresponding probabilities of the mosquito remaining infectious on day $j^\star$ since infectiousness onset, $j^\star = 1, \ldots, D^\star_{max}$, is denoted by $\gamma^\star_{j^\star} = P(D^\star \geq j^\star)$. Table S1 describes three sensitivity settings for the infectious period in mosquitoes: (1) the short setting has range 5 to 9 days with mean 7 days, (2) the medium setting has range 8 to 12 days with mean 10 days, and (3) the long setting has range 12 to 18 days with mean 15 days.

Given the assumed distributions above, we derive the relative infectivity of an infected case to an exposed susceptible person for a range of serial intervals. Intervals $k$ that are more likely have higher relative infectivity $\theta_k$. From the probabilities $\{\gamma_j : j = 1, \ldots, D_{max}\}$ and $\{\gamma^\star_{j^\star} : j^\star = 1, \ldots, D^\star_{max}\}$, we derive the relative infectivity for the interval $k$ between onset of an index case and onset of a secondary case as:

$$\theta_k = \sum_{j=1}^{D_{max}} \sum_{j^\star=1}^{D^\star_{max}} \gamma_j \gamma^\star_{j^\star} \mathbf{1}_{(j+j^\star+\delta^\star-1=k)}$$

for $k : \delta^\star + 1 \leq k \leq D_{max} + D^\star_{max} + \delta^\star - 1$, where $\delta^\star$ is the extrinsic latent period for infected mosquitoes. The $\theta_k$'s are then standardized to have mean one so that when we estimate the transmission probability (defined later) as $p(t)\theta_k$, $p(t)$ is interpreted as the average transmissibility over the infectious period. For simplicity, we use the same notation for the standardized $\theta_k$'s. At the bottom of Table S1, we report the exact values of the $\theta_k$'s for three combinations of the human and mosquito infectious period (short/short, medium/medium, long/long).

Table S1: *Natural history of ZVD.*

| Host Type | Period | Sensitivity Settings | Minimum | Maximum | Probability Mass | Mean |
|---|---|---|---|---|---|---|
| Human | Incubation | Short | 3 | 7 | (0.4, 0.2, 0.2, 0.1, 0.1) | 4.3 |
| | | Medium | 3 | 7 | (0.1, 0.2, 0.4, 0.2, 0.1) | 5 |
| | | Long | 3 | 7 | (0.1, 0.1, 0.2, 0.2, 0.4) | 5.7 |
| | Infectious | Short | 2 | 6 | Uniform | 4 |
| | | Medium | 4 | 8 | Uniform | 6 |
| | | Long | 6 | 10 | Uniform | 8 |
| Mosquito | Latent | Fixed | 10 | 10 | Fixed at 10 days | 10 |
| | Infectious | Short | 5 | 9 | Uniform | 7 |
| | | Medium | 8 | 12 | Uniform | 10 |
| | | Long | 12 | 18 | Uniform | 15 |
| Relative Infectiousness $\theta_k$ | | Short | 11 | 24 | (0.5, 1.0, 1.4, 1.7, 1.9, 1.9, 1.7, 1.42, 1.08, 0.7, 0.4, 0.2, 0.08, 0.02) | |
| | | Medium | 11 | 29 | (0.32 0.63, 0.95, 1.27, 1.52, 1.71, 1.84, 1.9, 1.84, 1.71, 1.52, 1.27, 0.96, 0.68, 0.44, 0.25, 0.13, 0.051, 0.013) | |
| | | Long | 11 | 37 | (0.23 0.45, 0.68, 0.9, 1.13, 1.35, 1.53, 1.67, 1.76, 1.8, 1.8, 1.8, 1.77, 1.7, 1.61, 1.48, 1.32, 1.13, 0.91, 0.7, 0.51, 0.35, 0.23, 0.13, 0.064, 0.026, 0.0064) | |

# 2 Estimation of effective reproductive numbers($R_t$) and basic reproductive number ($R_0$)

Suppose we observe an epidemic from day 1 to day $T$ among a population of $N$ individuals. We assume random mixing within the population. We assume the epidemic is initiated by a few imported cases in the early phase. Although case-importation is possible throughout the whole epidemic, it usually plays a negligible role for outbreaks of emerging infectious diseases in large populations. For this reason, we assume each susceptible person can be infected by any contact outside the study population with a probability of $b$ per day, but we fix $b$ at a extremely low value which is assumed known. Estimation of $b$ is often difficult and unreliable, if not impossible, for epidemic curve data. Let $p_0(t)$ be the baseline probability that a susceptible individual is infected by an infectious individual (via mosquito bites) on day $t$. To adjust for observed

covariates, the effective transmission probability is modeled via $\log \frac{p(t)}{1-p(t)} = \log \frac{p_0(t)}{1-p_0(t)} + \boldsymbol{x}'\boldsymbol{\beta}$ where $\boldsymbol{\beta}$ is the coefficient vector for covariate vector $\boldsymbol{x}$. For the two outbreaks in Colombia, the covariates considered are gender and three age groups (0-19, 20-49, and 50+). We let $p_0(t)$ be time-dependent to account for temporal heterogeneity in the virus's transmissibility caused by factors that are difficult to observe, for example, seasonality, intervention or changes in social contact behavior. To estimate effective reproductive numbers $R_t$, a usual practice is to partition the epidemic period into several intervals and to assume $p_0(t)$ is piece-wise constant over these intervals. To simplify notation, let $\boldsymbol{p}$ be the vector of transmission probabilities to be estimated. When $p(t)$ is constant over the epidemic, $\boldsymbol{p}$ is a scalar and is denoted by $p$.

**Transmission model** Let $\tilde{t}_i$ be the day of illness onset for person $i$, and let $\tilde{t}_i = \infty$ if $i$ is not infected by the end of the epidemic. Define $\tilde{\boldsymbol{t}} = \{\tilde{t}_i : i = 1, \ldots, N\}$ be the collection of observed symptom onset days in the population. The probability that a susceptible person $i$ escapes infection on day $t$ is given by

$$e_i(t) = (1-b)\prod_{j=1}^{N}\big(1 - \ \theta_{t-\tilde{t}_j+1} p(t) \mathbf{1}_{\tilde{t}_j \le t}\big). \tag{1}$$

from which the likelihood contribution of individual $i$ can be derived as

$$L_i(\boldsymbol{p}, \boldsymbol{\beta}) = \begin{cases} \prod_{t=1}^{T} e_i(t), & \tilde{t}_i = \infty, \\ \sum_{t=\tilde{t}_i-\delta_{max}}^{\tilde{t}_i-\delta_{min}} \alpha_{\tilde{t}_i-t}\big(1-e_i(t)\big)\prod_{\tau=1}^{t} e_i(\tau), & \tilde{t}_i < T. \end{cases} \tag{2}$$

To adjust for the selection bias that the outbreak is initiated by imported cases and that the exposure history of imported cases is unknown, imported cases do not contribute their own individual likelihoods, although their infectious periods do contribute to likelihoods of non-imported cases [6]. Let $\tilde{t}_0$ be the illness onset day of the last imported case. This adjustment also requires our likelihood calculation to start from $\tilde{t}_0 - \delta_{max} + 1$, which is the first possible day of infection for non-imported cases. We use the following adjusted likelihood

$$L_i^C(\boldsymbol{p}, \boldsymbol{\beta}) = \begin{cases} \prod_{t=\tilde{t}_0-\delta_{max}+1}^{T} e_i(t), & \tilde{t}_i = \infty, \\ \sum_{t=\tilde{t}_i-\delta_{max}}^{\tilde{t}_i-\delta_{min}} \alpha_{\tilde{t}_i-t}\big(1-e_i(t)\big)\prod_{\tau=\tilde{t}_0-\delta_{max}+1}^{t} e_i(\tau), & \tilde{t}_i < T, \end{cases} \tag{3}$$

which is a close approximation to the exact but much more complex adjustment used by Yang and colleagues [6]. Estimation is obtained by maximizing the joint likelihood for the population:

$$L^C(\boldsymbol{p}, \boldsymbol{\beta}) = \prod_{i=1}^{N} L_i^C(\boldsymbol{p}, \boldsymbol{\beta}). \tag{4}$$

**Calculation of effective reproductive numbers $R_t$ and the basic reproductive number $R_0$**

The effective SAR is the probability that an infected person infects an exposed susceptible during his infectious period, based on the effective transmission probability $p(t)$, which is calculated as

$$\text{SAR}(t) = 1 - \prod_{j=1}^{D_{max}+D^{\star}_{max}+\delta^{\star}-1} \big(1 - p(t-j)\,\theta_j\big). \tag{5}$$

Let $S(t)$ be the number of susceptible individuals at time $t$. The effective reproductive number is defined as $R_t = S(t)\text{SAR}(t)$, the average number of secondary infections an infected person can generate during his or her infectious period based on the transmission probability of the pathogen at time $t$. When the population is sufficiently large such that $S(t) \approx N$ for all $t$, we can use $R_t \approx N\text{SAR}(t)$. If $p(t) = p$ is time-invariant, Yang and colleagues [7] showed that, for a sufficiently large $N$ and a sufficiently small $p$, the chain-binomial model converges to a Poisson model where the number of new cases at each day has a Poisson distribution, and $N\text{SAR} = N\Big[1 - \prod_{j=1}^{D}\big(1 - p\ \theta_j\big)\Big]$ has the interpretation of the basic reproductive number $R_0$, which is the average number of individuals a typical infected person infects during his or her infectious period in a large, fully susceptible population. A useful result of this asymptotic argument is that, even if only the numbers of cases are reported but the size of the underlying susceptible population is unknown, one can create a large artificial population by assuming each observed case corresponds to $K$ uninfected individuals for some large $K$ ($\geq 50$ is large enough), and then apply the chain-binomial model to estimate $R_0$.

To estimate $R_t$, we adapt a sliding window approach by Cori and colleagues [1] to the chain-binomial model. Specifically, for each day $t$ during the epidemic, we define a one-week

window $[t-3, t+3]$, i.e., the epidemic period is partitioned into three intervals, with the middle interval equal to the window and the other two corresponding to $[1, t-4]$ and $[t+4, T]$. We then assume the transmission probability $p(t)$ is piece-wise constant over the three intervals, and the associated parameters are $\boldsymbol{p} = (p_1, p_2, p_3)$. The effective reproductive number at $t$ is then estimated by $\hat{R}_t = N\hat{p}_2$, where $\hat{\boldsymbol{p}} = (\hat{p}_1, \hat{p}_2, \hat{p}_3)$ are the maximum likelihood estimates based on (4). The confidence interval of $R_t$ can be obtained using standard delta methods.

The asymptotic argument requires a continuously growing epidemic, which implies that, for valid estimation of $R_0$, the chain-binomial model has to be applied only to the growth phase of the epidemic. On the other hand, reporting bias in the early phase of the epidemic, in particular under-reporting when a new disease is emerging, often inflates the estimate of $R_0$. The inflation is a much more severe problem for diseases with relatively long serial intervals such as vector-borne diseases, because a large number of cases has to be explained by a handful of initial cases. For example, when the chain-binomial model assuming constant transmission probability $p$ is applied to the growth phase of the Girardot outbreak of, the estimate of $R_0$ can be over 100 (results not shown)

To overcome this difficulty, we start from the estimation of effective reproductive numbers $R_t$ during the growth phase, defined as the period $[T_1 - \delta_{mean}, T_2 - \delta_{mean}]$, where $T_1$ is the day at which the epidemic starts to grow, $T_2$ is the peak day of the epidemic, and $\delta_{mean}$ is the mean duration of the incubation period. Reporting of Zika cases is assumed to be $10\%$ on or before the start of the growth phase and increases linearly for 2, 4, or 6 weeks until reaching $100\%$. The observed case numbers are rescaled according to the assumed reporting ratios for all analyses. $R_0$ is calculated as the median of the effective $R_t$ values during the growth phase. We choose the median as it is more robust to extreme values than the mean, so that our estimate is less affected by extremely high values of $R_t$ due to under-reporting at the beginning of the epidemic. The $95\%$ confidence interval (CI) for $R_0$ is the median of the $95\%$ CIs for the effective $R_t$ estimates;

this approach is not exact but is conservative because it assumes perfect correlation among all $R_t$ estimates during the growth phase.

**Evaluating goodness-of-fit** Following the approach of Yang and colleagues [6], we track the exposure history of each participant and calculate the probability of illness onset of this person on each day based on the fitted model, conditioning on the epidemic history up to the day before. This conditional probability can be interpreted as the model-predicted frequency of illness onset per person-day. For person $i$ and each day $t \leq \tilde{t}_i$, the model-fitted probability of illness onset is

$$\pi_i(t) = \sum_{\tau=t-\delta_{max}}^{t-\delta_{min}} \Big\{\big(1 - e_i(\tau)\big) \prod_{s=t-\delta_{max}}^{\tau-1} e_i(s)\Big\}\alpha_{t-\tau}).$$

For $t > \tilde{t}_i$, person $i$ is not susceptible anymore and should therefore not contribute to the expected frequency. For a given model, the aggregated expected frequencies $\sum_{i=1}^{N} \pi_i(t)$, can be plotted together with the observed onset frequencies to show goodness-of-fit. In addition, the illness status of each susceptible person-day is a Bernoulli random variable with probability $\pi_i(t)$. For a random-mixing homogeneous population (when individual-level covariates are not considered), $\pi_i(t)$ is identical for all $i$ satisfying $\tilde{t}_i \geq t$. Denote the common value of $\pi_i(t)$ by $\pi(t)$. Then, conditioning on the number of susceptible individuals $S(t)$ and the expected probability of illness onset $\pi(t)$ per person-day, the model-predicted distribution for the number of illness onsets on day $t$ is Binomial$\big(S(t), \pi(t)\big)$. The pair of symmetric $\alpha/2$ and $1 - \alpha/2$ quantiles form a conditional $(1 - \alpha) \times 100\%$ CI for the number of illness onsets on day $t$. In accordance with the sliding-window approach for estimating $R_t$, for each window $[t - 6, t]$, we pick the day that is $\mu_\delta$ days after the middle point of the window (day $t - 3$), where $\mu_\delta = \sum_{j=\delta_{min}}^{\delta_{max}} j\alpha_j$ is the mean incubation period. Denote this day by $\tau(t)$. The expected frequency, $\sum_{i=1}^{N} \pi_i(\tau(t))$, and associated conditional $(1 - \alpha)100\%$ confidence interval are then plotted against $\tau(t)$ for assessing the goodness-of-fit.

Table S2: **Estimates of $R_0$ for the outbreak of ZVD in San Andres, Colombia, for each combination of assumed natural history of Zika and assumed temporal pattern of under-reporting. The reporting ratio is assumed to increase linearly from 10% on and before September 30, 2015, to 100% in 2, 4 or 6 weeks. The estimation of $R_0$ is based on the median estimates of effective $R_t$ during the growth phase of the outbreak, September 25 to November 2, 2015.**

| Human | Human | Mean | Time 10% to 100% Reporting | | | | | |
|---|---|---|---|---|---|---|---|---|
| Infectious | Incubation | Serial | 2 Weeks | | 4 Weeks | | 6 Weeks | |
| Period | Period | Interval | Est. | 95% CI | Est. | 95% CI | Est. | 95% CI |
| Short | Short | 18.8 | 1.71 | (1.46, 2.01) | 1.24 | (1.04, 1.48) | 1.37 | (1.15, 1.63) |
| Short | Medium | 19.5 | 1.72 | (1.47, 2.01) | 1.19 | (1.02, 1.39) | 1.18 | (0.99, 1.42) |
| Short | Long | 20.2 | 1.71 | (1.42, 2.04) | 1.21 | (1.02, 1.42) | 1.09 | (0.92, 1.29) |
| Medium | Short | 21.3 | 1.93 | (1.57, 2.24) | 1.63 | (1.34, 1.99) | 2.25 | (1.91, 2.65) |
| Medium | Medium | 22.0 | 1.82 | (1.49, 2.12) | 1.41 | (1.15, 1.74) | 1.93 | (1.63, 2.29) |
| Medium | Long | 22.7 | 1.78 | (1.52, 2.07) | 1.25 | (1.00, 1.55) | 1.70 | (1.42, 2.04) |
| Long | Short | 24.8 | 1.83 | (1.54, 2.21) | 2.37 | (1.96, 2.86) | 3.35 | (2.87, 3.91) |
| Long | Medium | 25.5 | 1.75 | (1.50, 2.07) | 2.07 | (1.70, 2.52) | 2.90 | (2.47, 3.42) |
| Long | Long | 26.2 | 1.76 | (1.46, 2.09) | 1.80 | (1.46, 2.21) | 2.51 | (2.11, 2.99) |

## 2.1 $R_0$ results

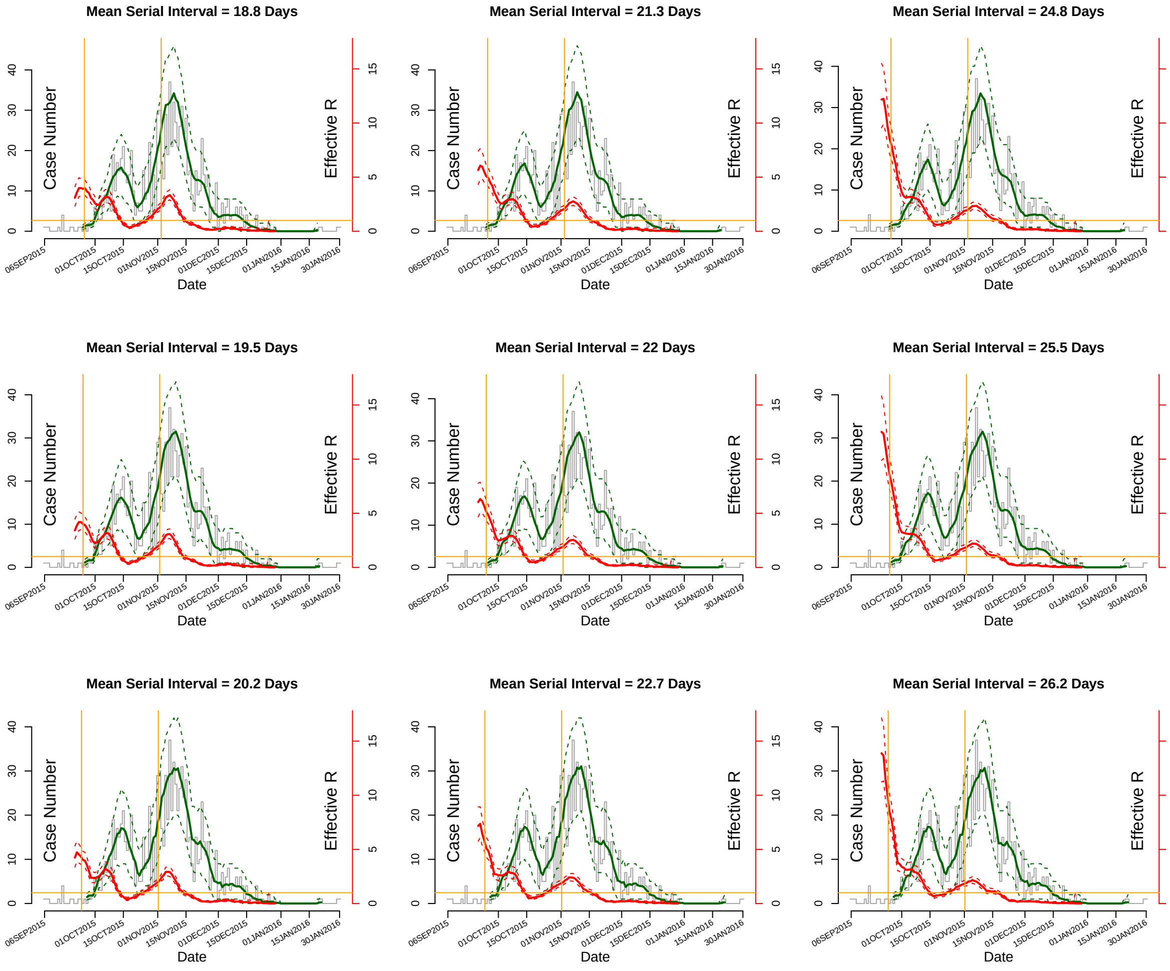


Figure S1: **Estimates of effective $R_t$ (red) and model-fitted daily case numbers (green) for the outbreak of ZVD in San Andres, Colombia, for each assumed natural history of Zika. The reporting ratio is assumed to increase linearly from 10% on and before September 30, 2015, to 100% in 2 weeks. Dashed curves (both red and green) are conservative 95% CIs. Histogram in grey shows the epidemic curve. The horizontal orange line indicates the reference value of 1. The two vertical orange lines indicate the time interval used for the estimation of $R_0$.**

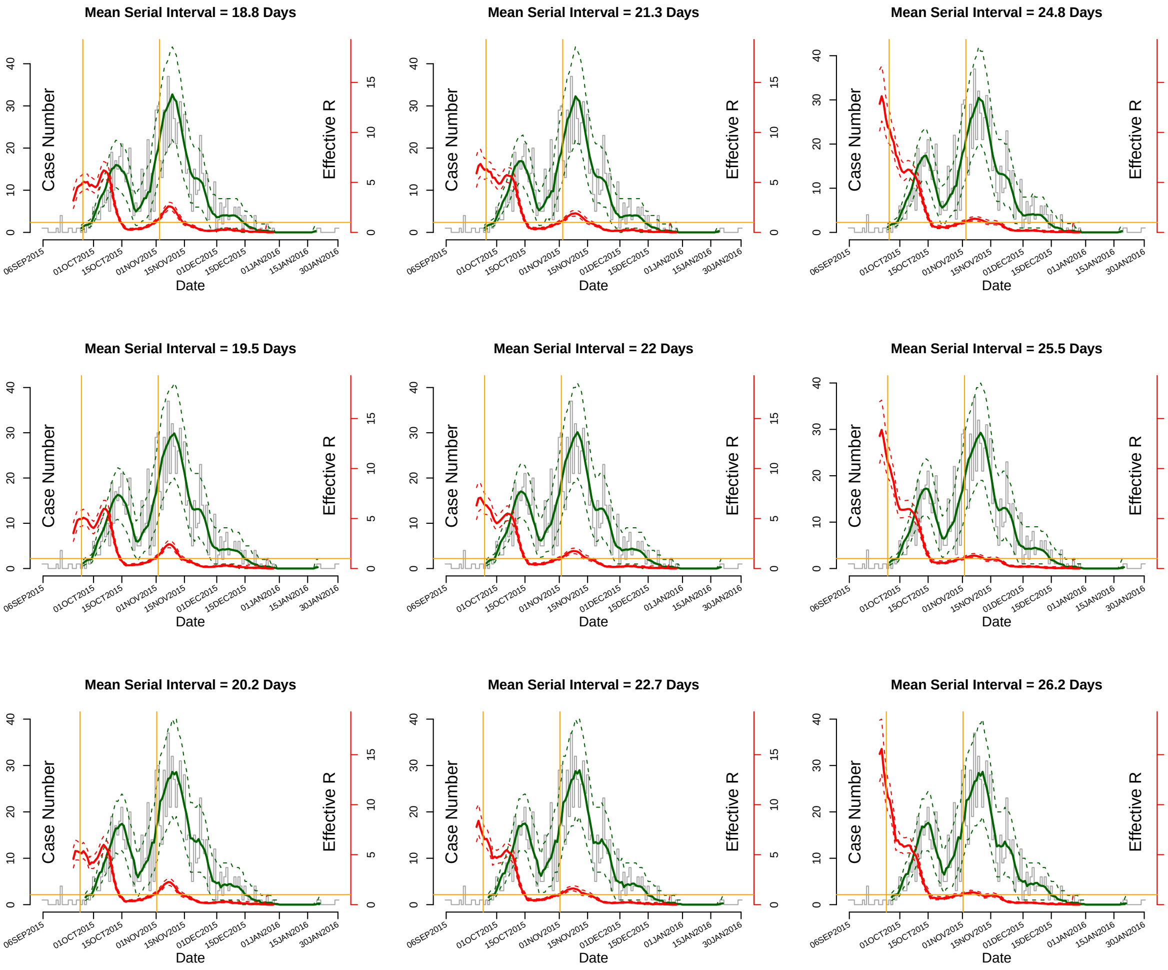


Figure S2: **Estimates of effective $R_t$ (red) and model-fitted dailycase numbers (green) for the outbreak of ZVD in San Andres, Colombia, for each combination of assumed natural history of Zika and assumed temporal pattern of under-reporting. The reporting ratio is assumed to increase linearly from 10% on and before September 30, 2015, to 100% in 4 weeks. Dashed curves (both red and green) are conservative 95% CIs. Histogram in grey shows the epidemic curve. The horizontal orange line indicates the reference value of 1. The two vertical orange lines indicate the time interval used for the estimation of $R_0$.**

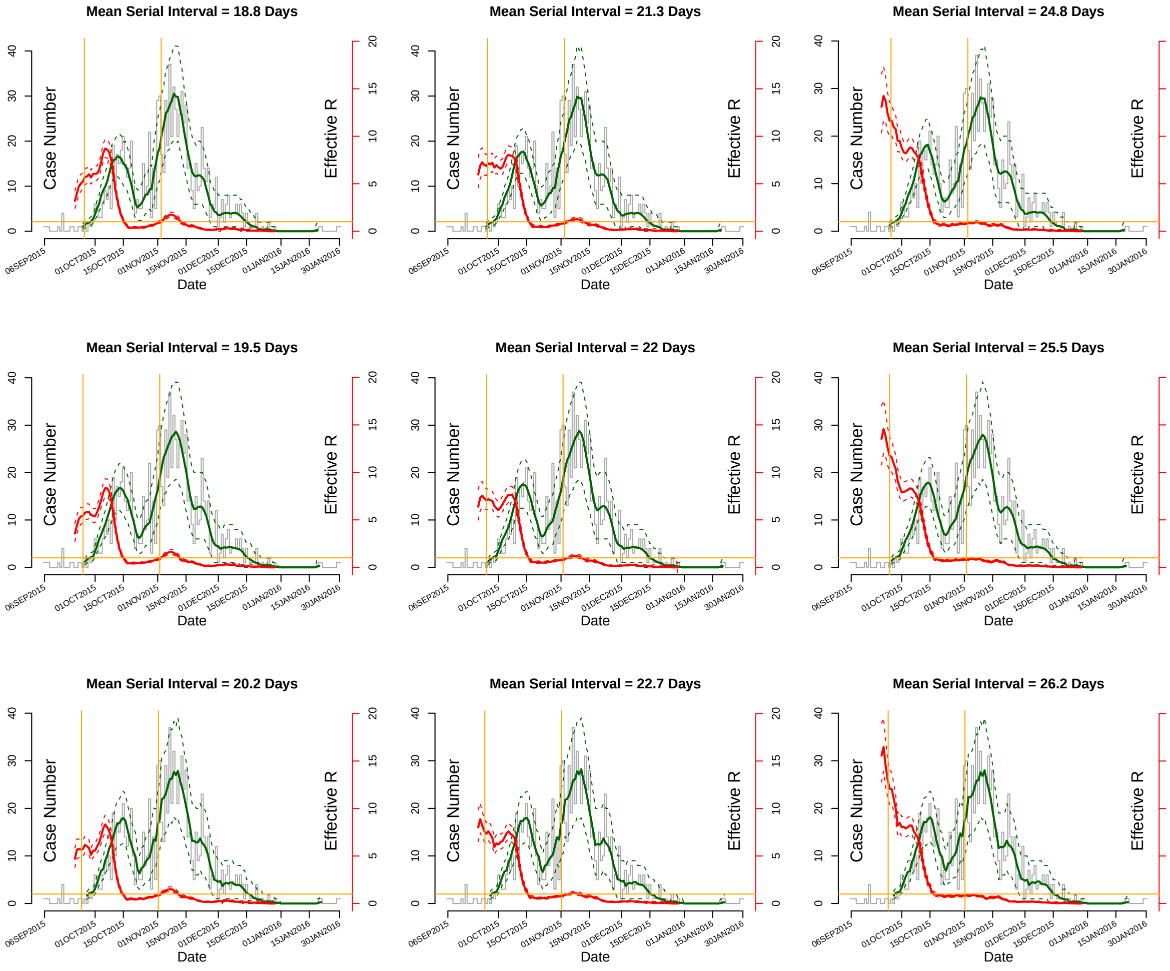


Figure S3: **Estimates of effective $R_t$ (red) and model-fitted daily case numbers (green) for the outbreak of ZVD in San Andres, Colombia, for each assumed natural history of Zika. The reporting ratio is assumed to increase linearly from 10% on and before September 30, 2015, to 100% in 6 weeks. Dashed curves (both red and green) are conservative 95% CIs. Histogram in grey shows the epidemic curve. The horizontal orange line indicates the reference value of 1. The two vertical orange lines indicate the time interval used for the estimation of $R_0$.**

Table S3: **Estimates of $R_0$ for the outbreak of ZVD in Girardot, Colombia, for each combination of assumed natural history of Zika and assumed temporal pattern of underreporting. The reporting ratio is assumed to increase linearly from 10% on and before October 19, 2015 to 100% in 2, 4 or 6 weeks. The estimation of $R_0$ is based on the median estimates of effective $R_t$ during the growth phase of the outbreak, October 19 to November 26, 2015.**

| Human | Human | Mean | Time 10% to 100% Reporting | | | | | |
|---|---|---|---|---|---|---|---|---|
| Infectious | Incubation | Serial | 2 Weeks | | 4 Weeks | | 6 Weeks | |
| Period | Period | Interval | Est. | 95% CI | Est. | 95% CI | Est. | 95% CI |
| Short | Short | 18.8 | 7.02 | (6.28, 7.85) | 3.65 | (3.25, 4.12) | 3.40 | (3.06, 3.77) |
| Short | Medium | 19.5 | 6.82 | (6.11, 7.61) | 3.64 | (3.25, 4.09) | 3.35 | (3.02, 3.71) |
| Short | Long | 20.2 | 6.54 | (5.85, 7.31) | 3.56 | (3.17, 3.99) | 3.21 | (2.90, 3.56) |
| Medium | Short | 21.3 | 8.84 | (7.92, 9.86) | 4.68 | (4.16, 5.26) | 4.42 | (3.99, 4.91) |
| Medium | Medium | 22.0 | 8.50 | (7.62, 9.48) | 4.61 | (4.11, 5.16) | 4.31 | (3.89, 4.77) |
| Medium | Long | 22.7 | 8.09 | (7.25, 9.03) | 4.48 | (4.00, 5.01) | 4.12 | (3.72, 4.56) |
| Long | Short | 24.8 | 13.23 | (11.89, 14.72) | 7.41 | (6.61, 8.30) | .7.29 | (6.60, 8.06) |
| Long | Medium | 25.5 | 12.65 | (11.37, 14.09) | 7.22 | (6.46, 8.07) | 7.04 | (6.37, 7.77) |
| Long | Long | 26.2 | 11.97 | (10.74, 13.34) | 6.97 | (6.24, 7.78) | 6.67 | (6.04, 7.36) |

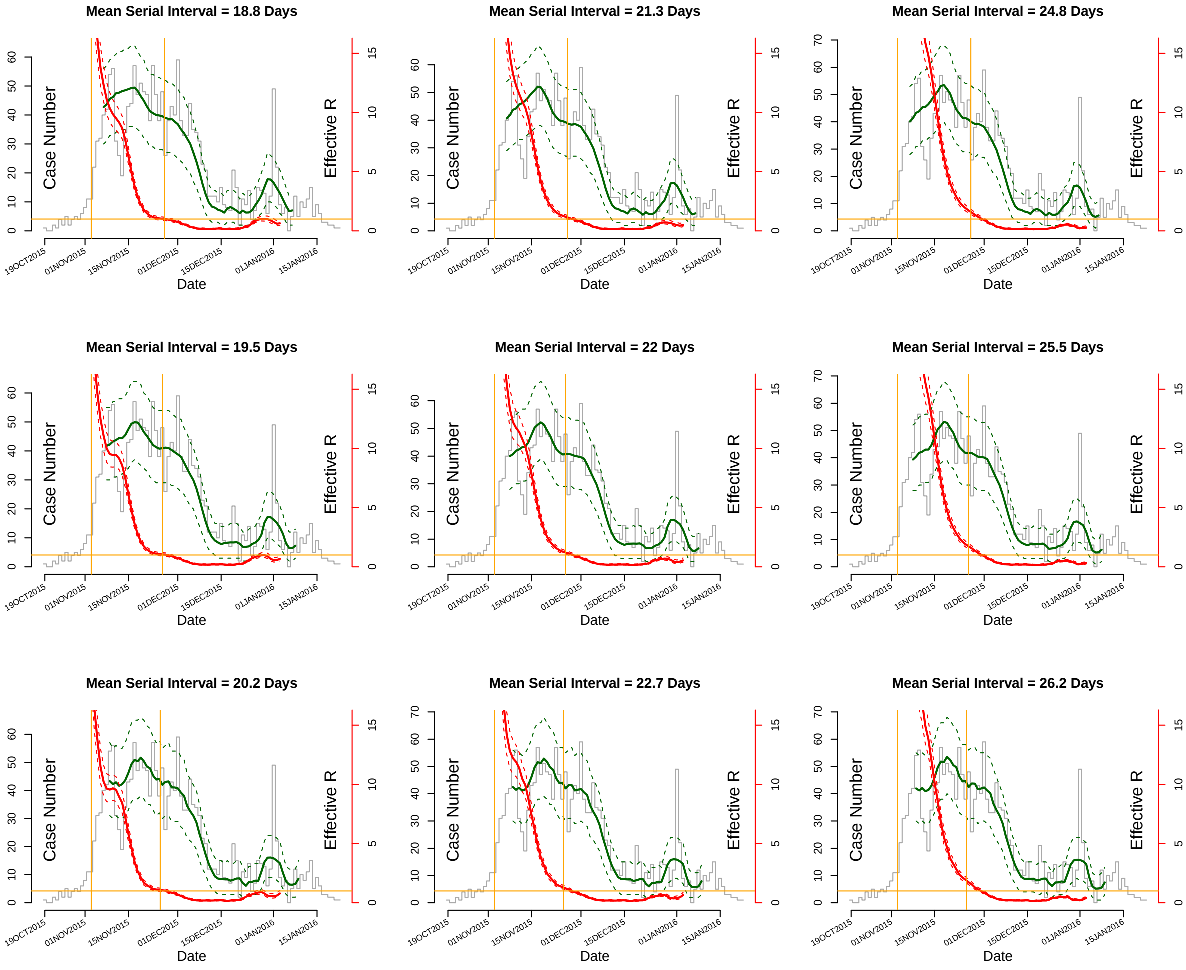


Figure S4: **Estimates of effective $R_t$ (red) and model-fitted daily case numbers (green) for the outbreak of ZVD in Girardot, Colombia, for each assumed natural history of Zika. The reporting ratio is assumed to increase linearly from 10% on and before October 19, 2015, to 100% in 2 weeks. Dashed curves (both red and green) are conservative 95% CIs. Histogram in grey shows the epidemic curve. The horizontal orange line indicates the reference value of 1. The two vertical orange lines indicate the time interval used for the estimation of $R_0$.**

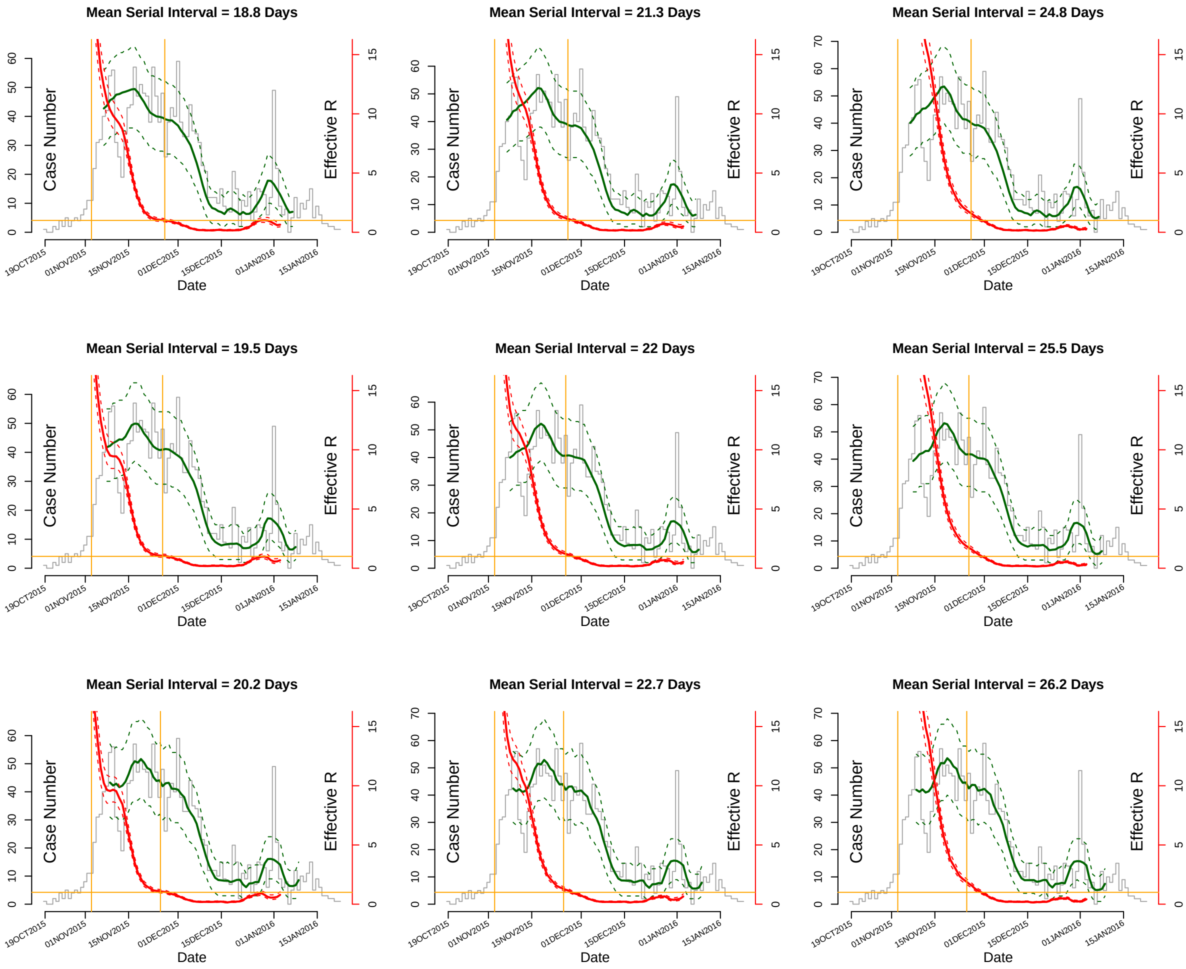


Figure S5: **Estimates of effective $R_t$ (red) and model-fitted daily case numbers (green) for the outbreak of ZVD in Girardot, Colombia, for each combination of assumed natural history of Zika and assumed temporal pattern of under-reporting. The reporting ratio is assumed to increase linearly from 10% on and before October 19, 2015, to 100% in 4 weeks. Dashed curves (both red and green) are conservative 95% CIs. Histogram in grey shows the epidemic curve. The horizontal orange line indicates the reference value of 1. The two vertical orange lines indicate the time interval used for the estimation of $R_0$.**

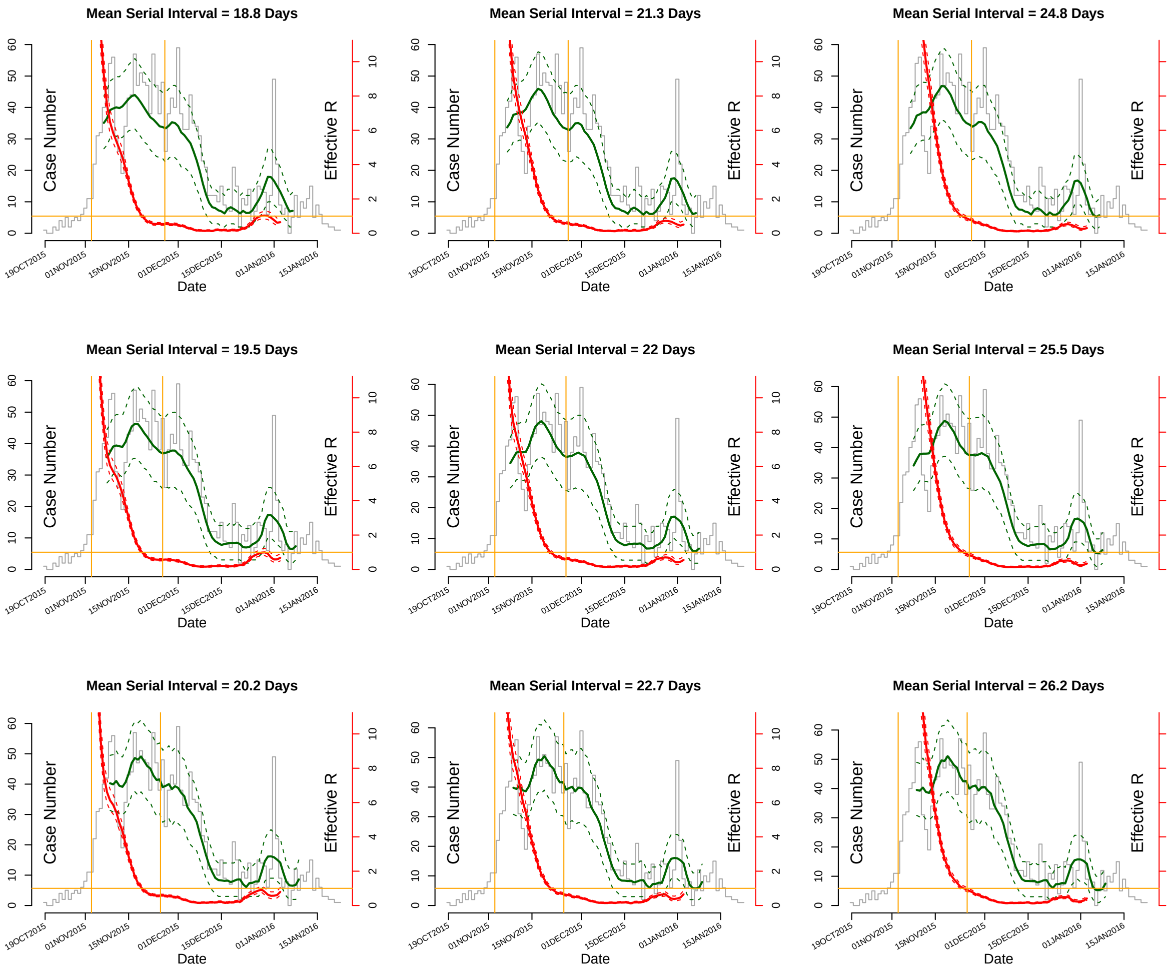


Figure S6: **Estimates of effective $R_t$ (red) and model-fitted daily case numbers (green) for the outbreak of ZVD in Girardot, Colombia, for each assumed natural history of Zika. The reporting ratio is assumed to increase linearly from 10% on and before October 19, 2015, to 100% in 6 weeks. Dashed curves (both red and green) are conservative 95% CIs. Histogram in grey shows the epidemic curve. The horizontal orange line indicates the reference value of 1. The two vertical orange lines indicate the time interval used for the estimation of $R_0$.**

Table S4: **Estimates of $R_0$ for the outbreak of ZVD in Salvador, Brazil, for each combination of assumed natural history of Zika and assumed temporal pattern of under-reporting. The reporting ratio is assumed to increase linearly from 10% on and before March 30, 2015, to 100% in 2, 4 or 6 weeks. The estimation of $R_0$ is based on the median estimates of effective $R_t$ during the growth phase of the outbreak, March 25 to May 3, 2015.**

| Human | Human | Mean | Time 10% to 100% Reporting | | | | | |
|---|---|---|---|---|---|---|---|---|
| Infectious | Incubation | Serial | 2 Weeks | | 4 Weeks | | 6 Weeks | |
| Period | Period | Interval | Est. | 95% CI | Est. | 95% CI | Est. | 95% CI |
| Short | Short | 18.8 | 1.22 | (1.14, 1.29) | 1.39 | (1.32, 1.47) | 1.71 | (1.62, 1.79) |
| Short | Medium | 19.5 | 1.24 | (1.16, 1.32) | 1.37 | (1.30, 1.45) | 1.71 | (1.63, 1.80) |
| Short | Long | 20.2 | 1.21 | (1.14, 1.29) | 1.39 | (1.32, 1.47) | 1.71 | (1.64, 1.80) |
| Medium | Short | 21.3 | 1.26 | (1.18, 1.35) | 1.42 | (1.34, 1.50) | 1.79 | (1.71, 1.88) |
| Medium | Medium | 22.0 | 1.28 | (1.20, 1.36) | 1.42 | (1.35, 1.49) | 1.81 | (1.73, 1.90) |
| Medium | Long | 22.7 | 1.27 | (1.19, 1.35) | 1.43 | (1.36, 1.50) | 1.86 | (1.76, 1.95) |
| Long | Short | 24.8 | 1.28 | (1.21, 1.35) | 1.47 | (1.38, 1.56) | 1.97 | (1.87, 2.06) |
| Long | Medium | 25.5 | 1.29 | (1.22, 1.36) | 1.45 | (1.37, 1.53) | 1.98 | (1.89, 2.06) |
| Long | Long | 26.2 | 1.26 | (1.19, 1.33) | 1.48 | (1.41, 1.56) | 2.02 | (1.93, 2.11) |

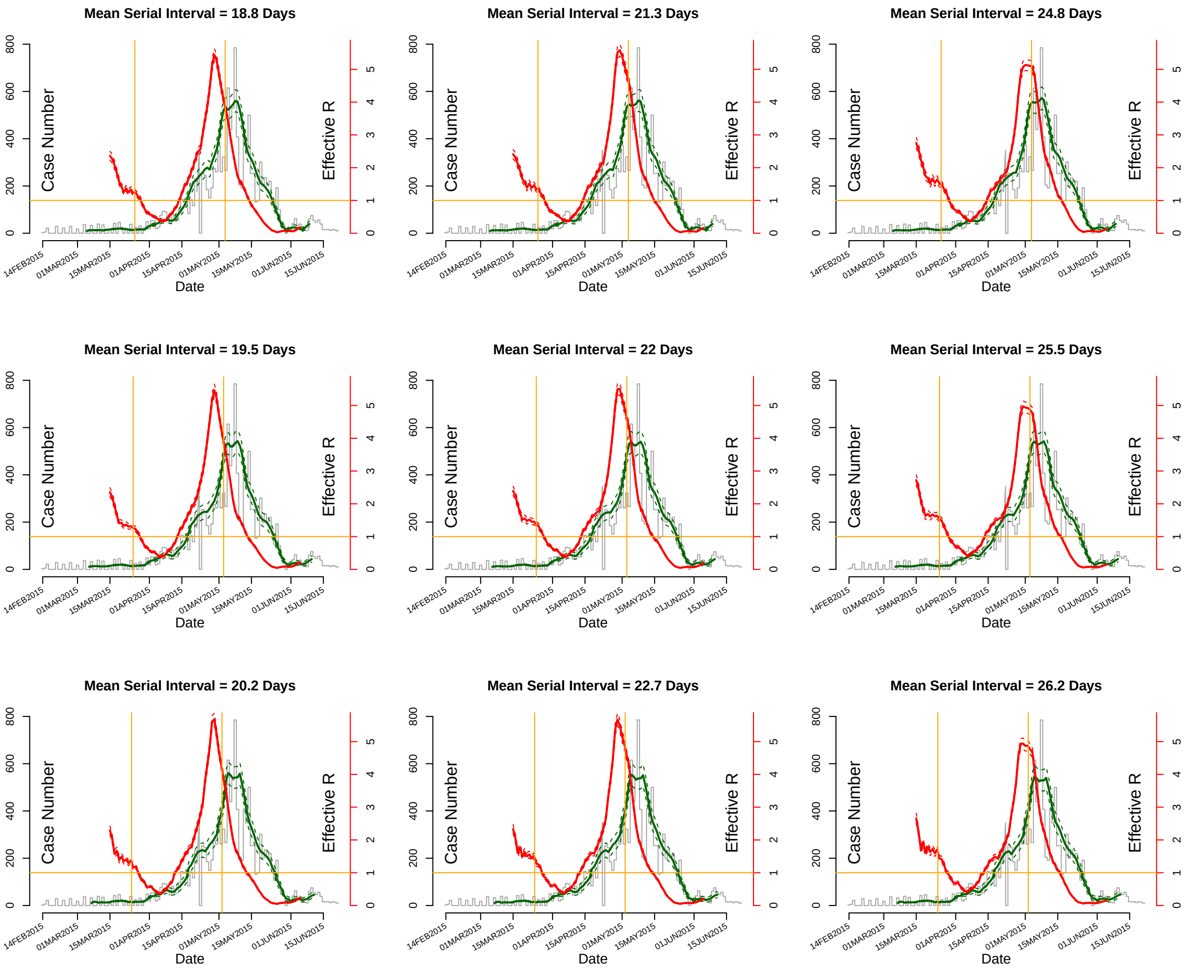


Figure S7: **Estimates of effective $R_t$ (red) and model-fitted daily case numbers (green) for the outbreak of ZVD in Salvador, Brazil, for each assumed natural history of Zika. The reporting ratio is assumed to increase linearly from 10% on and before March 30, 2015, to 100% in 2 weeks. Dashed curves (both red and green) are conservative 95% CIs. Histogram in grey shows the epidemic curve. The horizontal orange line indicates the reference value of 1. The two vertical orange lines indicate the time interval used for the estimation of $R_0$.**

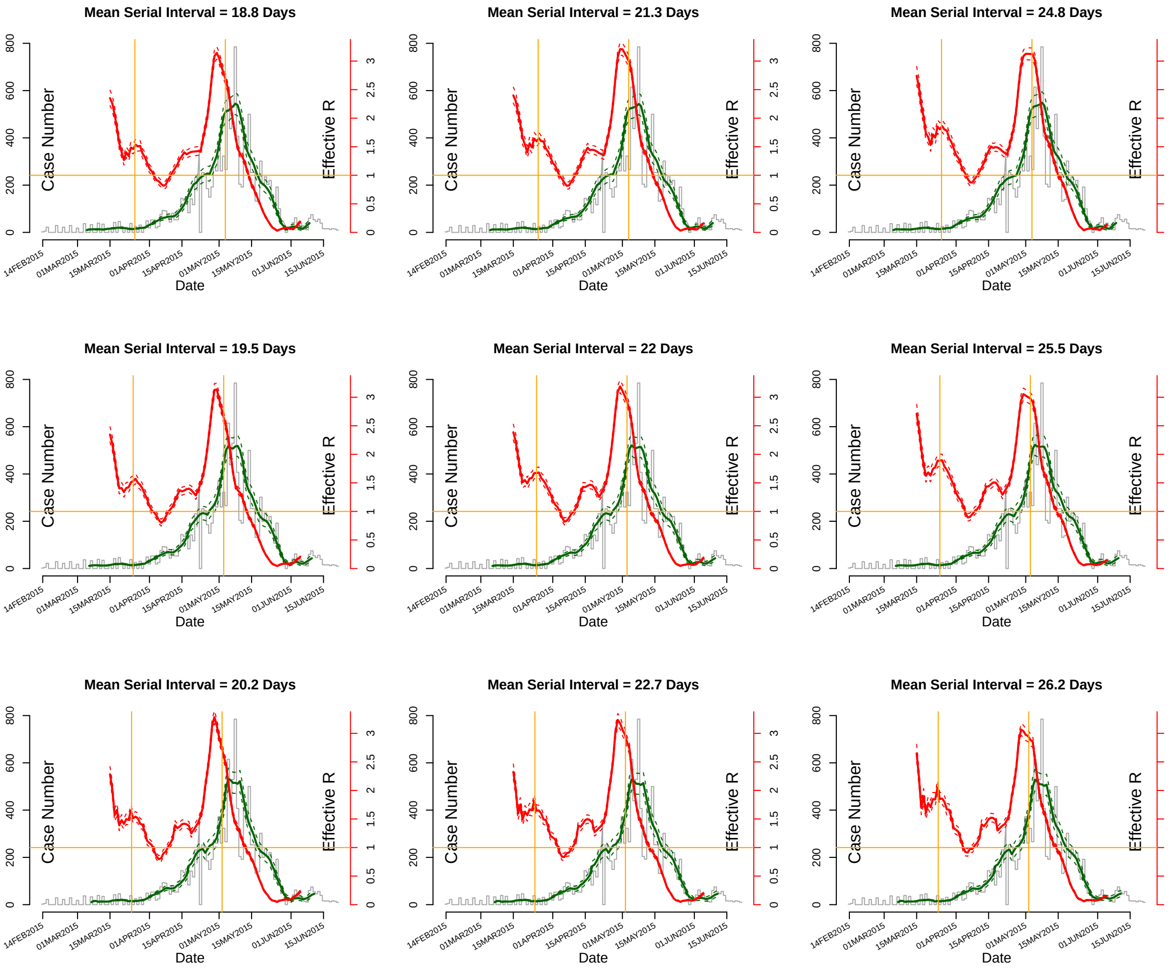


Figure S8: **Estimates of effective $R_t$ (red) and model-fitted daily case numbers (green) for the outbreak of ZVD in Salvador, Brazil, for each combination of assumed natural history of Zika and assumed temporal pattern of under-reporting. The reporting ratio is assumed to increase linearly from 10% on and before March 30, 2015, to 100% in 4 weeks. Dashed curves (both red and green) are conservative 95% CIs. Histogram in grey shows the epidemic curve. The horizontal orange line indicates the reference value of 1. The two vertical orange lines indicate the time interval used for the estimation of $R_0$.**

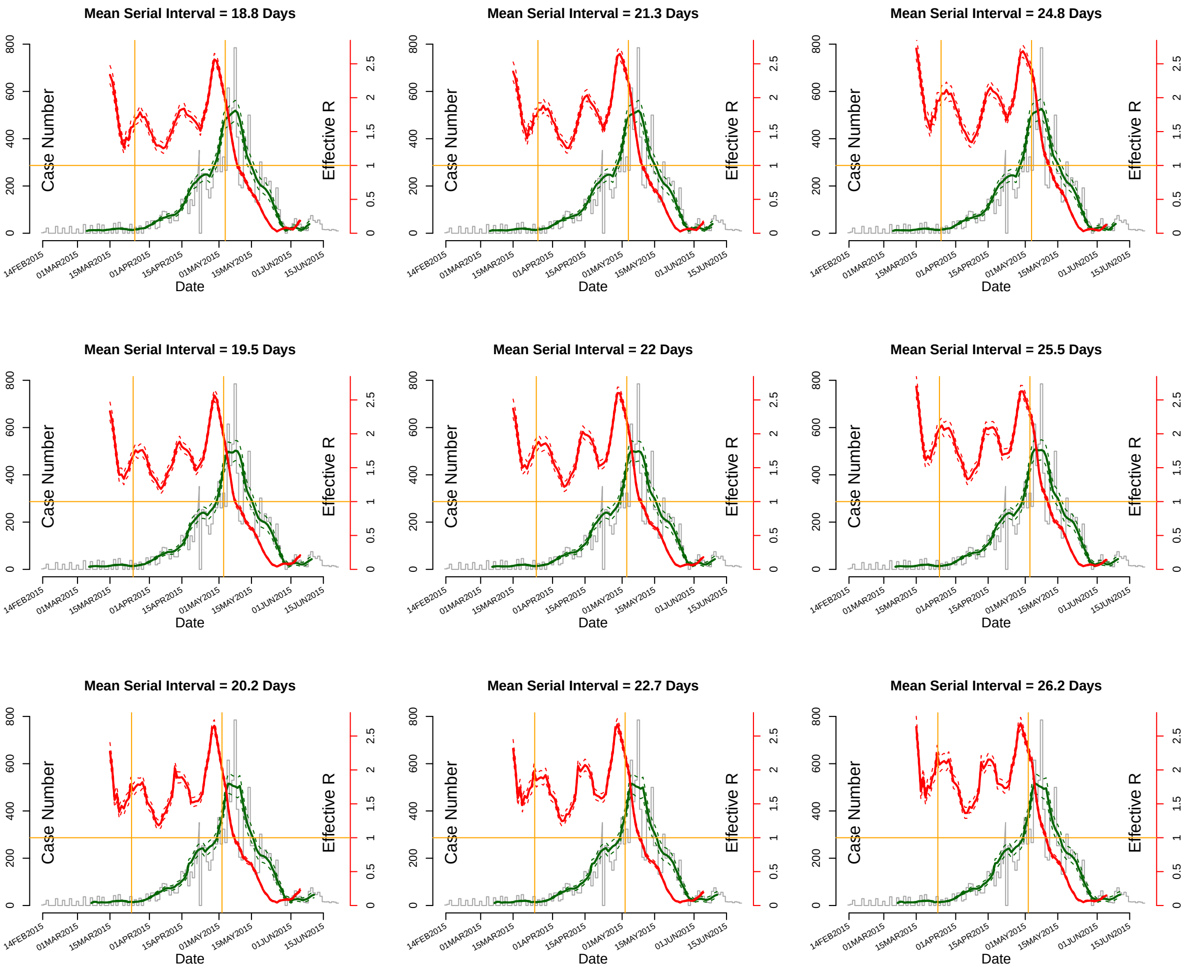


Figure S9: **Estimates of effective $R_t$ (red) and model-fitted daily case numbers (green) for the outbreak of ZVD in Salvador, Brazil, for each assumed natural history of Zika. The reporting ratio is assumed to increase linearly from 10% on and before March 30, 2015, to 100% in 6 weeks. Dashed curves (both red and green) are conservative 95% CIs. Histogram in grey shows the epidemic curve. The horizontal orange line indicates the reference value of 1. The two vertical orange lines indicate the time interval used for the estimation of $R_0$.**

## 2.2 Sensitivity analyses for under-reporting

Sensitivity analyses are performed assuming that reporting at the start of the outbreak is 30% rather than 10%. (See Figures S10 (San Andres), S11 (Girardot), and S12 (Salvador).)

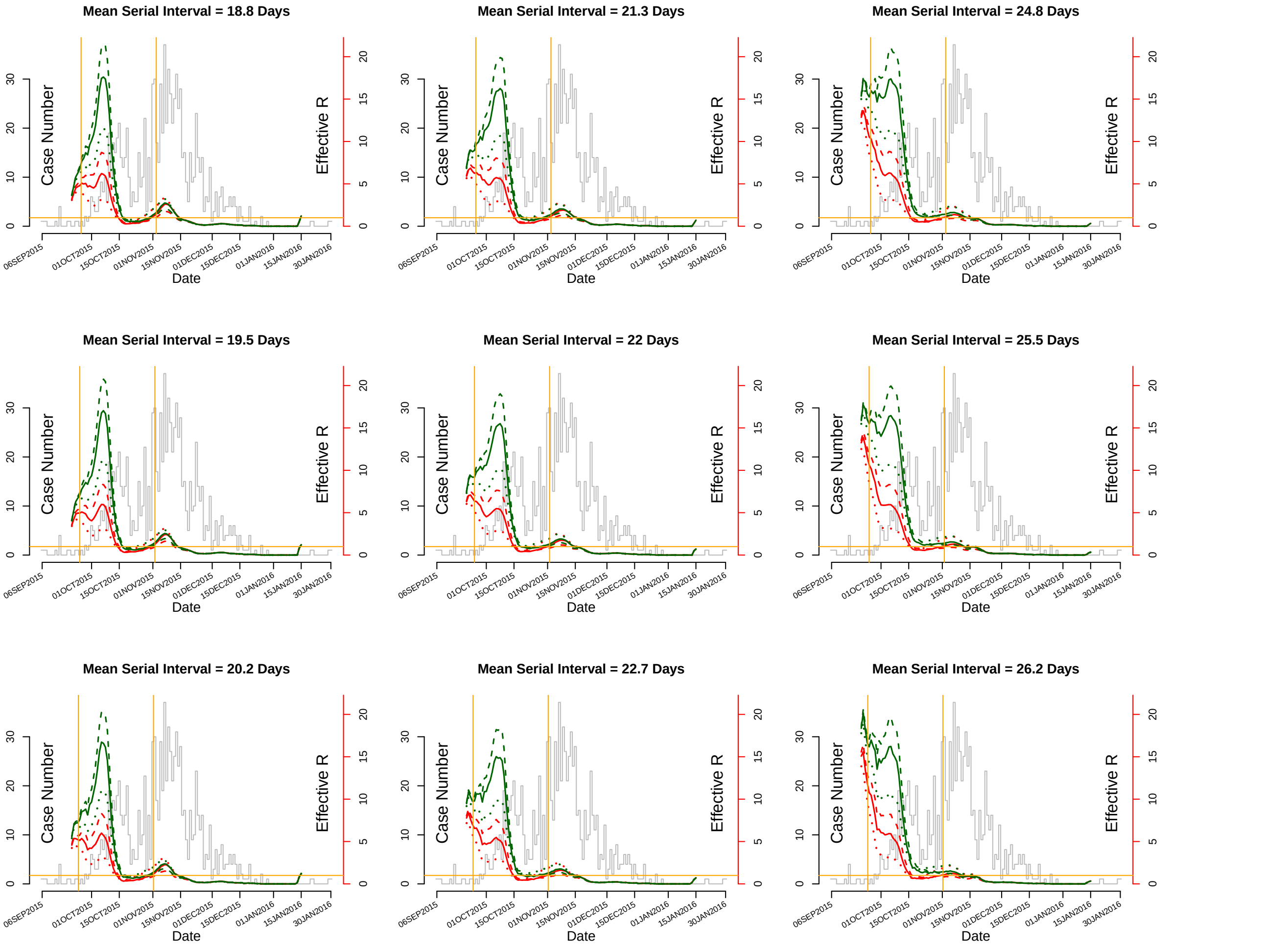


Figure S10: **Estimates of effective $R_t$ for the outbreak of ZVD in San Andres, Colombia,for each combination of assumed natural history of Zika and assumed temporal pattern of under-reporting. The reporting ratio is assumed to increase linearly from the baseline ratio of 10% (red) or 30% (green) on and before September 30, 2015, to 100% in 2 (dotted), 4 (dashed) or 6 (solid) weeks. Histogram in grey shows the epidemic curve. The horizontal orange line indicates the reference value of 1. The two vertical orange lines indicate the time interval used for the estimation of $R_0$.**

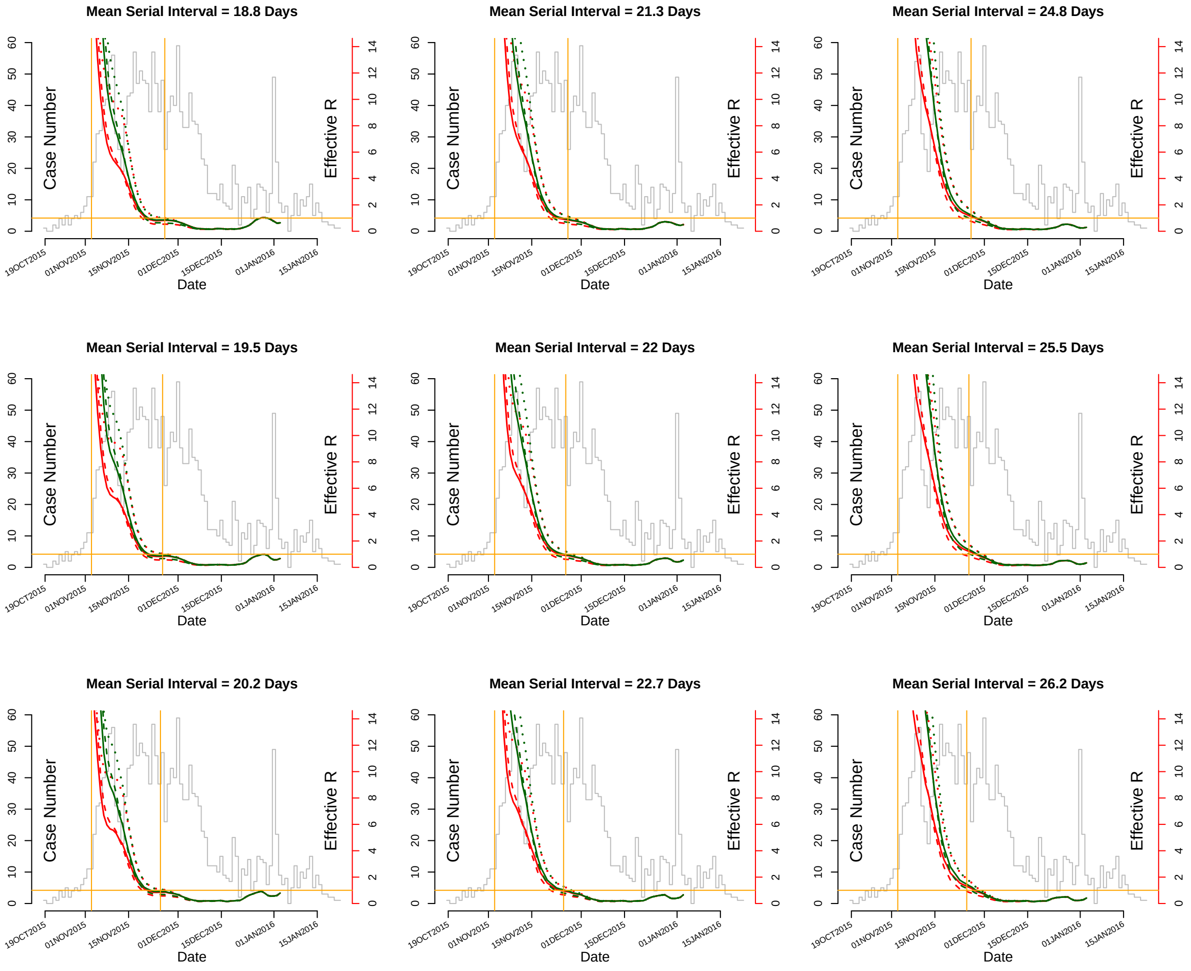


Figure S11: **Estimates of effective $R_t$ for the outbreak of ZVD in Girardot, Colombia, for each combination of assumed natural history of Zika and assumed temporal pattern of under-reporting. The reporting ratio is assumed to increase linearly from the baseline ratio of 10% (red) or 30% (green) on and before October 19, 2015, to 100% in 2 (dotted), 4 (dashed) or 6 (solid) weeks. Histogram in grey shows the epidemic curve. The horizontal orange line indicates the reference value of 1. The two vertical orange lines indicate the time interval used for the estimation of $R_0$.**

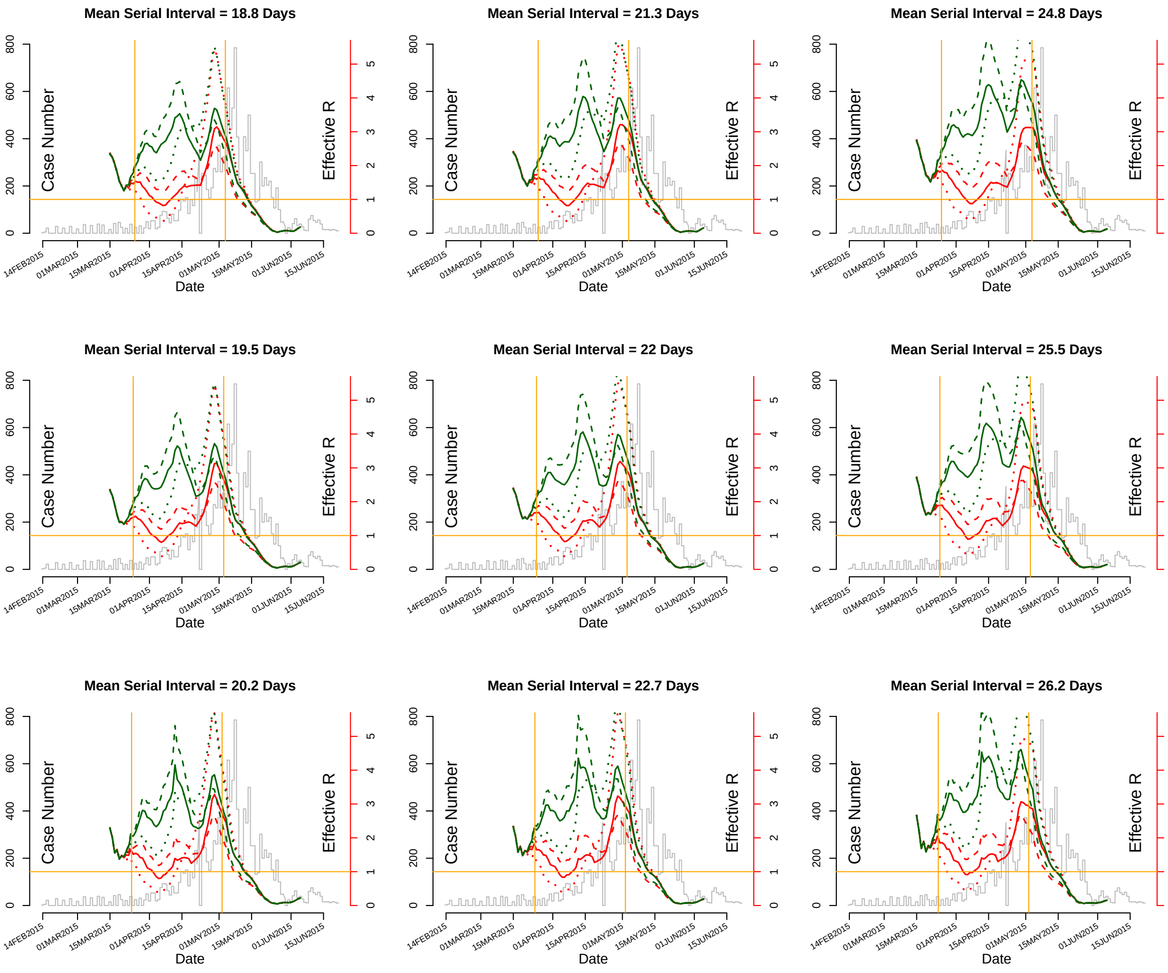


Figure S12: **Estimates of effective $R_t$ for the outbreak of ZVD in Salvador, Brazil, for each combination of assumed natural history of Zika and assumed temporal pattern of under-reporting. The reporting ratio is assumed to increase linearly from the baseline ratio of 10% (red) or 30% (green) on and before March 30, 2015, to 100% in 2 (dotted), 4 (dashed) or 6 (solid) weeks. Histogram in grey shows the epidemic curve. The horizontal orange line indicates the reference value of 1. The two vertical orange lines indicate the time interval used for the estimation of $R_0$.**

# References and Notes


[1] A. Cori, N. M. Ferguson, C. Fraser and S. Cauchemez. A new framework and software to estimate time-varying reproduction numbers during epidemics. *Am J Epidemiol*, 178(9):1505–1512, 2013.

[2] Jonathan F. Day, John D. Edman, and Thomas W. Scott. Reproductive fitness and survivorship of aedes aegypti (diptera: Culicidae) maintained on blood, with field observations from thailand. *Journal of Medical Entomology*, 31(4):611–617, 1994.

[3] European Centre for Disease Prevention and Control. Rapid risk assessment: Zika virus infection outbreak, French Polynesia.

[4] E. B. Hayes. Zika virus outside Africa. *Emerging Infectious Diseases*, 15(9):1347–1350, 2009.

[5] LINDA M. STYER, JAMES R. CAREY, JANE-LING WANG, and THOMAS W. SCOTT. Mosquitoes do senesce: Departure from the paradigm of constant mortality. *The American Journal of Tropical Medicine and Hygiene*, 76(1):111–117, 2007.

[6] Y. Yang, I.M. Longini, Jr., and M. E. Halloran. Design and evaluation of prophylactic interventions using infectious disease incidence data from close contact groups. *Applied Statistics*, 55:317–330, 2006.

[7] Y. Yang, J. D. Sugimoto, M. E. Halloran, N. E. Basta, D. L. Chao, L. Matrajt, G. Potter, E. Kenah, and I. M. Longini, Jr. The transmissibility and control of pandemic influenza A (H1N1) virus. *Science*, 326:729–733, 2009.
